# Oxidative Phosphorylation Does Not Violate the Second Law of Thermodynamics

**DOI:** 10.1021/acs.jpcb.4c03047

**Published:** 2024-08-21

**Authors:** Todd P. Silverstein

**Affiliations:** Department of Chemistry (emeritus), Willamette University, Salem, Oregon 97301,United States

## Abstract

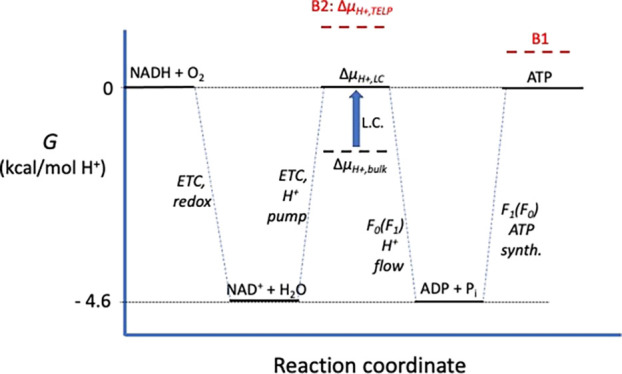

In a recent series of papers, James W. Lee reported that mitochondrial oxidative phosphorylation violates the second law of thermodynamics and that it is allowed to do so because it is a “Type-B” process that features lateral and longitudinal membrane asymmetry. We show here that these contentions are based on problematic interpretations of the literature. More reliable values of Δ*G*_redox_ and Δ*G*_ATP synthesis_ show that the second law is not violated. More recent reports on the structures of the redox-driven proton pumps (Complexes I, III, and IV) suggest that longitudinal membrane asymmetry does not exist. Finally, Lee’s predictions for the concentration of protons localized at the P-side surface of the bioenergetic membrane are likely to be much too high due to several errors; thus, his predicted high values of ΔpH_surface_ that violate the second law are likely to be wrong. There is currently no strong experimental or theoretical evidence to support the contention that oxidative phosphorylation violates the second law of thermodynamics.

## Introduction

In a recent series of papers, James Lee reported that oxidative phosphorylation in mitochondria violates the second law of thermodynamics in two distinct ways.^1−5^ Here, we wish to examine carefully the evidence and assumptions underlying this bold claim. We begin with a brief introduction to oxidative phosphorylation, which is currently understood in light of the chemiosmotic theory, for which Peter Mitchell won the 1978 Nobel Prize.^6^ Chemiosmotic ATP synthesis is driven by a transmembrane difference of the proton electrochemical potential, Δμ_H+_. Initially, Δμ_H+_ is established by redox-driven proton pumping by the electron transfer chain coupling sites. There are three thermodynamic components to this system: (i) energy input from spontaneous redox reactions catalyzed by the coupling sites (e.g., oxidation of NADH, FADH_2_); (ii) a high-energy intermediate, the proton electrochemical gradient, Δμ_H+_; and (iii) energy output via proton-driven ATP synthesis.

Δμ_H+_ comprises an electrical component, the transmembrane potential difference (Δψ), and a chemical component, the proton concentration gradient (ΔpH), as given by eq 1:
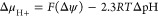
1

For our purposes, we will define the proton transport reaction in eq 1 as import from the outside (P) to the inside (N):^7^ Δψ ≡ ψ_*N*_ – ψ_*P*_ is invariably inside-negative, while ΔpH ≡ pH_N_ – pH_P_ is generally positive (i.e., inside-alkaline, except for alkaliphilic bacteria). Thus, Δμ_H+_(P → N) is invariably negative, proton import is spontaneous, and the free energy from proton influx can be used to drive nonspontaneous ATP synthesis.

Mitchell and many of the early proponents of the chemiosmotic theory insisted that the relevant proton concentrations were in the bulk aqueous phases on either side of the bioenergetic membrane: ΔpH_bulk_ ≡ pH_N,bulk_ – pH_P,bulk_. Studies on chloroplast thylakoid membranes, whose ATP synthase utilizes a high H^+^/ATP coupling ratio (4.67 = 14/3), supported this contention.^8^ However, as early as 1979, questions arose as to whether the energy in the bulk phase ΔpH was sufficient to drive ATP synthesis.^9,10^ Studies on alkaliphilic bacteria, which thrive at external pH values well above their cytoplasmic pH (making ΔpH_bulk_ negative, thus detracting from Δμ_H+_), showed that oxidative phosphorylation readily occurred even when the bulk phase Δμ_H+_ was insufficient to drive ATP synthesis.^11−14^ Similar concerns were raised regarding low-potential bacteria and mitochondria, which utilize lower H^+^/ATP coupling factors of 3.3 and 2.7.^8^ Recently, high-precision narrowly focused measurements of mitochondrial pH have shown that ΔpH_bulk_ across the mitochondrial F_1_F_0_ ATP synthase (≈0.07 to 0.32 pH units^15−21^) is even lower than previously reported (0.85–1.1 pH units). It has been suggested that this low ΔpH may yield a Δμ_H+_ that is insufficient to drive mitochondrial ATP synthesis.^15,22^

To explain these thermodynamically problematic results, many bioenergeticists hypothesized that proton concentration at the outer (P) surface of the bioenergetic membrane exceeded that in the bulk phase; in other words, the bulk and surface phases were maintained out of equilibrium, because protons localized at the membrane surface were not easily released into the bulk phase. This hypothesis (ΔpH_surface_ > ΔpH_bulk_) has been dubbed “localized chemiosmosis”. The lack of equilibration has been explained by two quite different models: Lee’s protonic capacitor model^1,23^ and the potential well/barrier model.^24−27^

**Figure 1:**
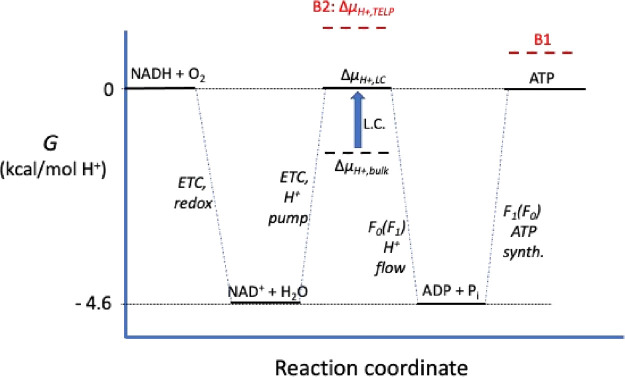
Free energy profile of redox-driven proton pumping and proton-driven ATP synthesis. L.C. = localized chemiosmosis: the bulk Δμ_H+_ is insufficient to drive ATP synthesis, requiring a higher, localized ΔpH_surface_. NADH and FADH_2_ oxidation yield ≈ −4.6 kcal/mol H^+^, which is identical to the energy required for proton-flow driven ATP synthesis in the matrix. B = type-B processes that violate the 2nd law of thermodynamics because the redox energy input is exceeded by (B1) ATP synthesis energy (output) and/or (B2) Δμ_H+,TELP_ due to high ΔpH_surface_ established by a “protonic capacitor”.

In the past few years, James W. Lee has published a series of papers^1−5^ suggesting that oxidative phosphorylation violates the second law of thermodynamics in two different ways: (1) The energy required for ATP synthesis (final output) exceeds that supplied by redox reactions (initial input, dotted red B1 line in Figure 1), and also, (2) the energy in the surface-localized high-energy intermediate Δμ_H+,TELP_ exceeds the redox energy input (dotted red B2 line in Figure 1). According to Lee, because of asymmetric structural aspects of the bioenergetic membrane, environmental heat energy can be harnessed to help synthesize ATP. Lee refers to this as a “thermotrophic” feature of a “type-B” process, which is not constrained by the second law.

A free energy profile depicts the three thermodynamic components of oxidative phosphorylation (Figure 1). The left-most reaction, NADH (or FADH_2_) oxidation, releases a maximum of ≈4.6 kcal per mole of protons pumped (Supporting Information, section IIA).^8,22^ Protons flowing back across the membrane through the F_0_ oligomer supply the 4.6 kcal/mol H^+^ (=12.3 kcal/mol ATP ÷ 2.67 H^+^/ATP) required for ATP synthesis in the matrix (Supporting Information, section IIB). The overall maximum thermodynamic efficiency^a^ of the process in the matrix is ≈100%; for ATP exported to the cytoplasm, the maximum thermodynamic efficiency is 91% (15.4 kcal/mol ATP ÷ 3.67 H^+^/ATP ÷ 4.6 kcal/mol H^+^(redox); Supporting Information, section IIB). All processes obey the second law of thermodynamics: redox energy input ≥ Δμ_H+,LC_ ≥ ATP synthesis energy output.

In the free energy profile (Figure 1), the need for localized chemiosmosis (L.C.) is depicted by the black dashed line segment: Here, the bulk-to-bulk Δμ_H+_ is insufficient to drive ATP synthesis. Lee’s two type-B processes are depicted by the two red dashed line segments, wherein redox energy input is exceeded by B1, ATP synthesis energy, and/or B2, the localized Δμ_H+,TELP_.

Claiming that oxidative phosphorylation violates the second law of thermodynamics is a bold contention. Oxidative phosphorylation, which first appeared in bacteria over 2 billion years ago, is a ubiquitous metabolic process in eukaryotic mitochondria and prokaryotic cells. It seems surprising at first glance that such a universal process could violate such a widely embraced thermodynamic law. We have therefore undertaken to examine the evidence supporting Lee’s claim.

## Results and Discussion

### B1: ATP Synthesis Requires More Energy than Redox Reactions Can Supply

In making this claim, Lee reported^2,3,28^ Δμ_H+_ = −5.26 kcal/mol H^+^ available from redox-driven proton pumping, but +5.85 kcal/mol H^+^ required for proton-driven ATP synthesis.^b^ Thus, the per proton energy input exceeds output, and the second law of thermodynamics^c^ is violated. It turns out, however, that both of these numbers are too high. The value of −5.26 kcal/mol H^+^ available from redox-driven proton pumping is derived from standard redox potentials and thus applies to the reactions carried out when all reactant and product concentrations (except for H^+^) are 1 M. We have reported previously that under typical steady state concentrations of aqueous NADH, NAD^+^, FAD, FADH_2_, and O_2_, Δμ_H+_ = −4.45 and −4.68 kcal/mol H^+^ for oxidation of NADH and FADH_2_, respectively.^8^ (See the Supporting Information, section IIA, for further details.)

Lee calculated +5.85 kcal/mol H^+^ required for proton-driven ATP synthesis by dividing Δ*G*_p_ = +15.6 kcal/mol ATP from Cockrell et al.^29^ by the H^+^/ATP coupling factor of 2.67 for the F_1_F_0_-catalyzed ATP synthesis in the mitochondrial matrix. However, Cockrell et al.’s value of Δ*G*_p_ was not in fact measured in the matrix of normally functioning intact state 3 mitochondria (see the Supporting Information, section IIB). A comprehensive meta-analysis of reported Δ*G*_p_ values (section IIC, Supporting Information) shows that its value in the mitochondrial matrix, where ATP is synthesized on the F_1_ complex, is 12.3 ± 0.8 kcal/mol ATP. Dividing this by 2.67 H^+^/ATP, we get Δμ_H+_ = +4.6 kcal/mol H^+^ flowing through the F_1_F_0_ ATP synthase. Note that this value is 21% lower than that used by Lee (5.85 kcal/mol H^+^) and is approximately equal to the available redox free energy (−4.6 kcal/mol H^+^). Considering ATP that is synthesized in the matrix and exported to the cytoplasm, we have Δ*G*_p,cytopl._ = 15.4 kcal/mol ÷ 3.67 H^+^/ATP = 4.2 kcal/mol H^+^ (see the Supporting Information, section IIB). Thus, the second law is not violated: Spontaneous redox reactions supply enough free energy to drive ATP synthesis.

### B2: The Surface-Localized Proton Gradient Exceeds Redox Energy Input

#### Calculating Lee’s Surface-Localized Proton Chemical Potential, Δμ_H+,P,L_

**Figure 2:**
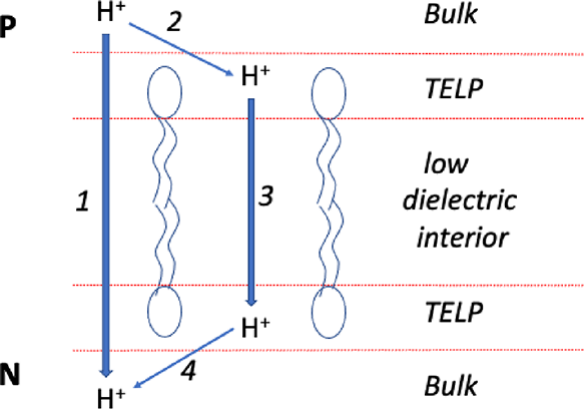
Proton transport steps in Lee’s model of transmembrane proton transport.

Considering spontaneous proton influx (P → N) through the F_0_ complex that drives ATP synthesis, Lee distinguished between the chemical potential differences for three distinct proton transport processes (Figure 2): (1) from P bulk to N bulk phase, Δμ_H+,bulk_(P → N), which is given by Mitchell’s eq 1; (2) from P bulk to the P surface-localized TELP layer, Δμ_H+,P,L_; and (3 + 4) the total for localized (TELP) chemiosmosis from the P surface to the N bulk phase, Δμ_H+,LC_(P → N).

Since P_bulk_ → N_bulk_ transport (Δμ_H+,bulk_(P → N)) is equivalent to the sum of P_bulk_ → P_TELP_ (Δμ_H+,P,L_) and P_TELP_ → N_bulk_ (Δμ_H+,LC_(P → N)), we have
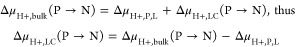
2

Lee derived the following definition for Δμ_H+,P,L_:
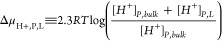
3

According to Lee, the proton concentration in the P surface layer = [H^+^]_P,bulk_ + [H^+^]_P,L_, where [H^+^]_P,L_ is the concentration of “excess” protons localized in the P surface TELP layer due to the TELP protonic capacitor electrostatic effect. From eq 3, we see that as long as [H^+^]_P,L_ > 0, Δμ_H+,P,L_ is positive; furthermore, as discussed above, due to redox-driven proton pumping, Δμ_H+,bulk_(P → N) is negative. Thus, we see from eq 2 that Δμ_H+,LC_(P → N) is more negative than Δμ_H+,bulk_(P → N), with the difference determined by the magnitude of Δμ_H+,P,L_: That is, more free energy is released in localized chemiosmosis than is available in the bulk phase gradient alone.

There are several serious problems with the thermodynamic and structural biological assumptions used by Lee in his derivations of eqs 2 and 3 above. These are discussed in detail in section III of the Supporting Information. However, for the purposes of our discussion here, we will accept the validity of these equations. In order to calculate Δμ_H+,P,L_ (and thus the net Δμ_H+,LC_(P → N)), Lee used the following electrostatic model to calculate the concentration of “excess” localized protons, [H^+^]_P,L_.

#### Lee’s TELP Protonic Capacitor Equations

Since his first paper^30^ on this subject in 2012, Lee has characterized the localization of protons at the membrane surface using his transmembrane electrostatically localized protons (TELP) hypothesis. In this model, Lee assumes that a bioenergetic membrane that supports a nonzero transmembrane potential functions like a capacitor in an electrical circuit. Protons, because they diffuse much faster than other ions,^d^ behave like electrons in a circuit. Two TELP surface layers that stretch 1 nm from either side of the low-dielectric membrane interior out toward the aqueous phase serve as the two plates of the “protonic capacitor”. Beyond the 1 nm-thick TELP layer lies the bulk phase. Excess protons at the P surface, and hydroxide anions at the N surface, are held within the TELP layers due to strong electrostatic attraction.^e^ Using electrostatics equations describing electronic capacitors, Lee derived:
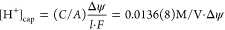
4where *C* = capacitance, *A* = area, *l* = thickness of the surface layer, and *F* = Faraday’s constant. Lee used *l* = 1 nm, as a “reasonable” thickness, with no explanation, and *C*/*A* = 13.2 mF/m^2^ = 1.32 μF/cm^2^, which was measured across leukemia cell plasma membranes.^32^ The multiplying factor, *C*/*(A·l·F*), then comes out to be 0.0136(8) M/V.

There are problems with Lee’s selected values for both *C*/*A* and *l. C*/*A* values are clearly higher for cancer cells (1.2–2.1 μF/cm^2^) than for normal cells and organelles (0.5–1.2 μF/cm^2^, Supporting Information, section V). Thus, Lee’s choice of 1.32 μF/cm^2^ is undoubtedly too high. The value reported^33^ for neuronal plasma membranes, 0.94 ± 0.20 μF/cm^2^ (range: 0.8–1.3 μF/cm^2^), seems like a reasonable choice for bioenergetic membranes. Furthermore, Lee’s choice of *l* = 1 nm supposedly represents the thickness of the bilayer headgroup region along with a monolayer of surface water at the headgroup/bulk interface (Lee, personal communication). Given that the lipid headgroup region is 1.1 ± 0.2 nm thick,^34−37^ and proton diffusion at the membrane surface involves 1–2 water monolayers at the headgroup/bulk interface,^38,27,26,25^ which would be about 0.4 nm thick, a better choice for the thickness of the TELP layer would be 1.5 nm. Using *l* = 1.5 nm and *C*/*A* = 0.94 μF/cm^2^, the multiplying factor, *C*/*(A·l·F*), comes out to be 0.0062(7) M/V, which is less than half of the value used by Lee.

Lee has pointed out that the actual [H^+^]_P,L_ is lower than [H^+^]_cap_ due to cations in solution exchanging with protons in the TELP surface layer. If the equilibrium constant for the M_i_^z+^ cation replacing protons in the L layer (H^+^_L_ + M_i_^z+^_bulk_ → H^+^_bulk_ + M_i_^z+^_L_) is *K*_exchg,i_, then considering *n* different cations in solution, *i* = 1 to *n*, Lee derived:
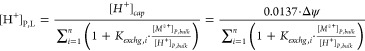
5

Lee used literature values for bulk concentrations of Na^+^, K^+^, Mg^2+^, Ca^2+^, Zn^2+^, Fe^2+^, and other cations. He used *K*_exchg_ values of 5.07(10^–8^) for Na^+^, 6.93(10^–8^) for K^+^, ≈6(10^–8^) for NH_4_^+^, and 2.1(10^–7^) for Mg^2+^ and all other divalent cations.^f^ Using these values together with literature values of mitochondrial P side bulk concentrations of the cations, Lee calculated the denominator in eq 5, dubbed the “exchange factor,” to be 1.29. There are reasons to believe that this value is orders of magnitude too low (see footnote f and Table 1).

**Table 1:** Key Thermodynamic Parameters Calculated from TELP Equations, Given pH_N,bulk_ = 7.35 and pH_P,bulk_ = 7.25^a^

**Δ****ψ****(mV)**	**[H**^**+**^**]**_**P,L**_, (mM)	**Δμ**_**H+,bulk**_**(P → N)**(kcal/mol H^±^)	**Δμ**_**H+,P,L**_(kcal/mol H^±^)	**Δμ**_**H+,LC**_**(P → N)**(kcal/mol H^±^)
–81	8.65	–2.0	7.5	–9.5
–123	13.1	–3.0	7.8	–10.74
–159	17.0	–3.8	7.9	–11.7
–180	19.2	–4.3	8.0	–12.3
–81*	1.7(10^–7^)	–2.0	0.144	–2.15
–123*	2.6(10^–7^)	–3.0	0.145	–3.1
–159*	3.3(10^–7^)	–3.8	0.1455	–3.9(5)
–180*	3.7(10^–7^)	–4.3	0.145	–4.4(4)

a The exchange factor (eq 5 denominator) is 1.29 in the top four rows (following Lee) and 6.6(10^7^) in the bottom four starred rows.

Lee used eq 5 to calculate the concentration of “excess” protons in the surface-localized TELP layer, [H^+^]_P,L_, as a function of Δψ, and then, using eqs 2 and 3, he calculated Δμ_H+,LC_(P → N). The range of Δψ that he employed was generally 50 to 200 mV, especially citing low literature values ranging from 56 to 114 mV. However, in vivo measurements suggesting a very low Δψ (−81 to −120 mV) have been challenged as experimental artifacts (Supporting Information, section IV).

In the Supporting Information (section IV), we present an extensive meta-analysis of reported Δψ values measured in mitochondria. The least negative reliable reported values are −123 mV, the most negative = −180 mV, and the average = −159 ± 16 mV (inside negative; Supporting Information, section IV). Lee selected bulk phase pH values of 7.25 on the P side and 7.35 on the N side; using eqs 2 and 5, we can calculate TELP predictions for key thermodynamic parameters under low-, average-, and high-potential conditions. Using Lee’s eq 5 denominator of 1.29 (Table 1, top four rows), the localized Δμ_H+,P,L_ comprises most (65–79%) of the net Δμ_H+,LC_(P → N).

Furthermore, as Lee has noted, even at the most negative observed Δψ value, Δμ_H+,bulk_(P → N) is less negative than the 4.6 kcal/mol H^+^ required for ATP synthesis; on the other hand, the total Δμ_H+,LC_(P → N) far exceeds the required ATP synthesis energy even for the least negative Δψ. This is the crux of Lee’s TELP hypothesis, namely, that TELP-predicted values of Δμ_H+,LC_(P → N) exceed the required ATP synthesis energy for all observed values of Δψ. In his recent papers,^1−4^ Lee has pointed out that Δμ_H+,P,L_ (and therefore, Δμ_H+,LC_(P → N)) exceeds the available redox energy input of −4.6 kcal/mol H^+^; hence, TELP predicts violation of the second law of thermodynamics.

There are two problems with the Δμ_H+,P,L_ and Δμ_H+,LC_(P → N) values in the top four rows of Table 1: (i) They are predicted values and have not been observed experimentally, and (ii) they are based on an ion exchange factor of 1.29, which in turn is based on ion exchange *K*_eq_ values that have been shown to be thermodynamically untenable^40,39^ and are undoubtedly orders of magnitude too low.

One obvious problem with ion exchange *K*_eq_ values of ≈10^–8^ is that the sizes of the hydrated cations do not differ greatly: radii in Å = 2.80/H^+^, 3.31/K^+^, 3.58/Na^+^, 4.12/Ca^2+^, 4.28/Mg^2+^.^41^ Considering only electrostatic forces, one would expect membrane surface affinity from the strongest to weakest to be H^+^> Ca^2+^ > Mg^2+^ > K^+^ > Na^+^. However, based on ionic size and charge, the differences should be less than 2 orders of magnitude, not the 7 or 8 orders of magnitude that Lee uses. Indeed, using more reasonable *K*_exchg_ values of 0.001 for Na^+^, 0.0015 for K^+^, and 0.006 for Mg^2+^, the denominator in eq 5, 6.6(10^7^), is more than 50 million times larger than 1.29. This in turn gives predicted [H+]_P,L_ values 50 million-fold smaller than those calculated by Lee (Table 1). The minuscule localized Δμ_H+,P,L_ = 0.14 kcal/mol yield Δμ_H+,LC_(P → N) values that are barely larger than Δμ_H+,bulk_(P → N); this, in turn, means that Lee’s calculated Δμ_H+,LC_(P → N) is insufficient to drive ATP synthesis (i.e., ≤4.6 kcal/mol H^+^).

Thus, all three of the parameters that Lee uses in eqs 4 and 5 are suspect: His *C*/*A* value of 1.32 μF/cm^2^ is too high by ≈45%, his arbitrarily selected TELP layer thickness of 1 nm is too low by ≈33%, and his M^+^/H^+^ ion exchange equilibrium constants are several orders of magnitude too low. This makes Lee’s calculated values of [H^+^]_P,L_ and TELP/surface-localized free energy highly suspect. Using reasonable ion exchange equilibrium constant values of ≈10^–3^ gives very low (≈0.3 μM) localized [H^+^]_P,L_; this yields a localized Δμ_H+,LC_(P → N) that does not violate the second law of thermodynamics but is also insufficient to drive ATP synthesis. On the other hand, using Saeed and Lee’s untenable ion exchange equilibrium constant values of ≈10^–8^ gives high (≈10 mM) localized [H^+^]_P,L_; this yields a localized Δμ_H+,LC_(P → N) that is sufficient to drive ATP synthesis but violates the second law. Lee’s response has been to embrace this second law violation, proposing that due to structural and topological asymmetries in the bioenergetic membrane, the establishment of the P surface-localized proton layer is a so-called “Type-B” process that is allowed to violate the second law of thermodynamics.^1−4^ He claimed, for example, “that transmembrane electrostatically localized excess protons at the liquid water–membrane interface can isothermally utilize their molecular thermal motions to do useful work in driving ATP synthesis at the biophysical molecular scale”.^1^

### TELP Experimental Support

#### Measurements of Surface pH

Using his TELP hypothesis, Lee has predicted low values of pH_P,surface_ (≈2). We list in Table S3 (Supporting Information, section VI) experimentally measured values of surface pH within 1 nm of the water/hydrophobic interface measured or calculated at eight different interfaces; the median surface pH for all 18 reported values is 5.4 ± 0.4 (±one standard deviation). Based on the range of reported values, we may conclude that the surface pH lies between ≈4.5 and 6.5. This is dramatically less acidic than the pH ≈ 2 values predicted by Lee’s TELP hypothesis; thus, literature reports do not support the predictions of the TELP hypothesis.

Lee has compared his TELP-predicted Δμ_H+,LC_(P → N) or protonmotive force (pmf) values to two experimental results reported in the literature: the doubling time of alkaliphilic *Bacillus pseudofirmus* OF4 bacteria^13^ and the ATP efflux rate through the mitochondrial adenine nucleotide translocase (ANT).^18^

#### TELP and Alkaliphilic Bacterial Cell Doubling

**Figure 3:**
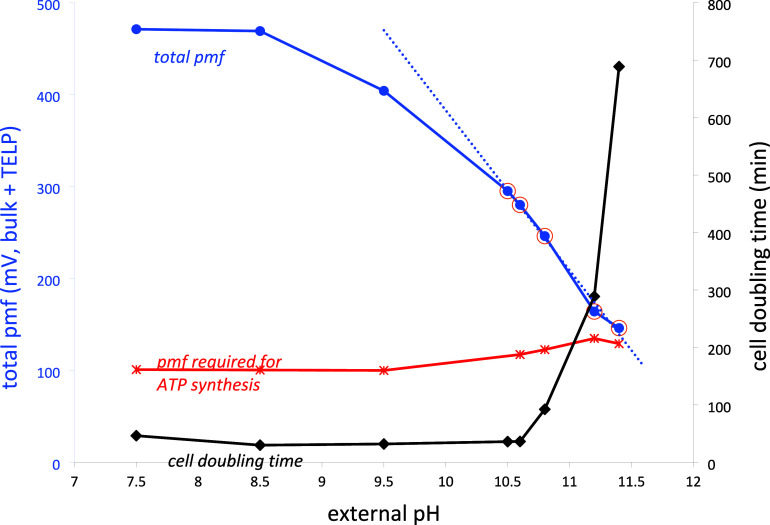
TELP-predicted total pmf (bulk + localized; blue circles, left-hand *y*-axis), pmf required for ATP synthesis (red squares, left-hand *y*-axis), and *B. pseudofirmus* OF4 cell doubling time (black diamonds, right-hand *y*-axis) as a function of external pH. Calculated total pmf from ref (1); ADP phosphorylation potential (in mV) and OF4 cell doubling time from ref (13). Linear decline in total pmf above pH 10.5: slope = −174 mV/pH unit; intercept = 2123 mV; *R*^2^ = 0.992.

Lee supported the bioenergetic significance of his TELP-predicted total protonmotive force (pmf) in alkaliphilic bacteria, writing the following: “the overall pattern of the total pmf [= -Δμ_H+,LC_(P → N)/*F*] amazingly matched with the *B. pseudofirmus* OF4... cell population growth doubling time... This indicates that we really are on something [sic] real”.^1^ However, we can see from the actual data plotted in in Figure 3 that the cell doubling time (black diamonds) remains stable (at 30–40 min) up to an external pH of 10.6; above that pH, doubling time rises exponentially toward infinity (i.e., no measurable doubling time above pH ≈ 11.5). On the other hand, total pmf (blue circles) is stable (at 470 mV), only up to external pH 8.5; above that pH, total pmf declines gradually and then linearly (pH ≥ 10.5). Thus, Lee’s claim of an “amazing” match between the pH-dependence of total pmf and cell doubling time is not supported.

Another problematic observation is that at pH 10.8, the cell doubling time is more than twice that at pH 10.6, and yet the TELP-calculated total pmf is more than twice that required for ATP synthesis (red squares, Figure 3). In fact, TELP-calculated total pmf exceeds that required for ATP synthesis up to the highest pH tested. Thus, the relationship between the TELP-calculated total pmf and that required for ATP synthesis is completely decoupled from the cell doubling time. In other words, the dependence of cell doubling time on external pH does not support the bioenergetic significance of the TELP total pmf.

**Figure 4:**
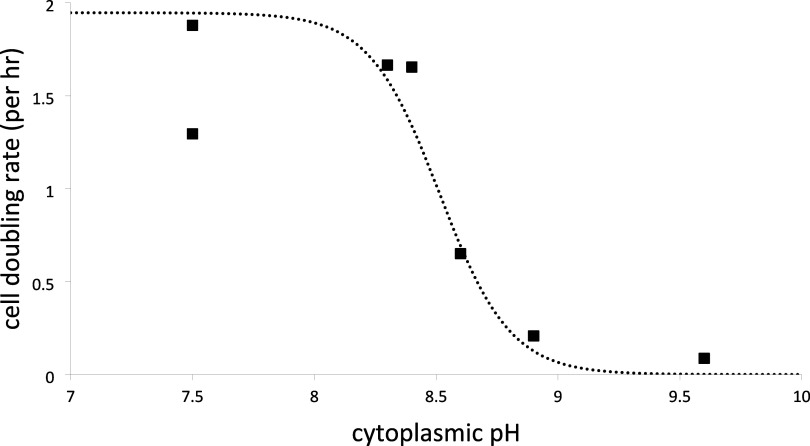
*B. pseudofirmus* OF4 cell doubling rate as a function of cytoplasmic pH. Data are adapted from ref (13). Points are fit by nonlinear regression (dotted curve) to the equation for a pH titration; best-fit values are p*K*_a_ = 8.51 ± 0.03; maximum rate = 1.95 ± 0.07 h^–1^; *n* = 2.9 ± 0.4; and *R*^2^ = 0.991.

It is interesting to note that regarding cell doubling, the rate (e.g., per hour) is a more appropriate parameter to consider than the time. Also, one would expect the cell doubling rate to be more sensitive to cytoplasmic pH than to external pH. Accordingly, Figure 4 shows that the cell doubling rate falls with the deprotonation of three (*n* = 2.9 ± 0.4) titratable acidic groups of p*K*_a_ = 8.51 ± 0.03. We have shown previously^22^ that the ATP synthesis rate in starved/respiration-inhibited *Bacillus firmus* OF4 cells^12^ energized solely by K+/valinomycin-induced Δψ ≈ −180 mV also falls with the deprotonation of titratable groups, p*K*_a_ = 8.6 to 8.8. Guffanti and Krulwich hypothesized^12^ that this pH dependence of ATP synthesis rate was due to a protonation-dependent gating residue. Perhaps similar (or identical?) gating residues control cell doubling rate as well.

#### TELP and Mitochondrial ATP Synthesis and Efflux

**Figure 5:**
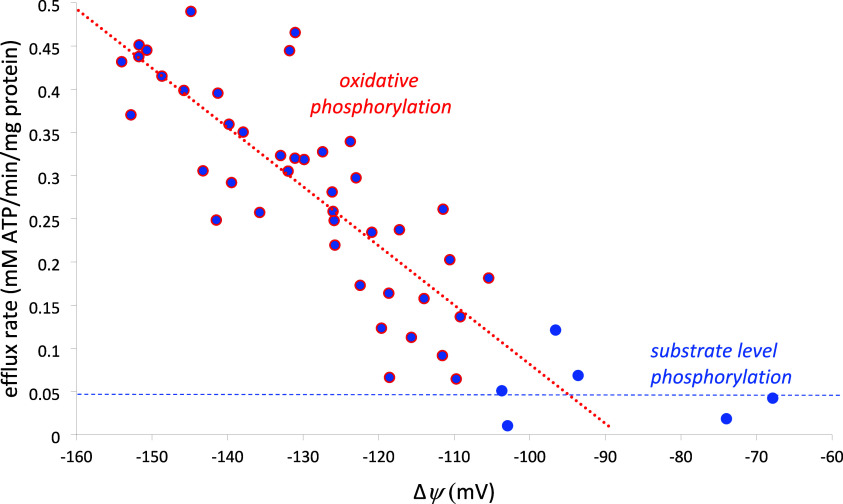
ATP-ADP steady-state exchange rate mediated by the ANT with Δψ clamped at various matrix pH values (6.8–7.9). Data from Figure 7C of ref (18). For Δψ ≥ −104 mV, the efflux rate falls to a constant value of 0.05 ± 0.04 mM ATP/min/mg protein; for Δψ ≤ −104 mV, the efflux rate declines linearly with increasing (i.e., less negative) Δψ: slope = −6.9 ± 0.8 μM ATP/min/mg protein per mV; *x*-intercept = −88 ± 18 mV; *R*^2^ = 0.66.

Lee supported the bioenergetic significance of his TELP-predicted total pmf in mitochondria, writing the following: “the observed pattern of the ATP efflux rate [through the ANT], which decreases as mitochondrial membrane potential (Δψ) is reduced, is generally also in agreement with the pattern of the total pmf [predicted from TELP equations]”.^3^ Although it is true that both the TELP-predicted total pmf and the ANT-mediated ATP efflux rate (Figure 5) decline roughly linearly as Δψ increases (i.e., gets less negative), there are some important differences.

There are two different types of mitochondrial ATP synthesis: oxidative phosphorylation is Δψ-dependent, whereas substrate level phosphorylation is Δψ-independent. As pointed out by Lee, substrate level phosphorylation generally supplies <10% of ATP under optimal aerobic conditions. This can be seen in Figure 5, where the blue dotted line at 0.05 ± 0.04 mM ATP/min/mg protein shows the substrate level phosphorylation at low potential (Δψ ≥ −104 mV, six points), and the red dotted line shows oxidative phosphorylation increasing linearly as Δψ gets more negative, from −105 to −155 mV. The important thing to note is that oxidative phosphorylation reaches zero at −95 mV, where the blue and red dotted lines intersect in Figure 5. However, Lee’s Figure 1 shows that the TELP-predicted total pmf greatly exceeds the pmf required for ATP synthesis even at Δψ = −50 mV. Thus, according to Lee’s TELP, ATP synthesis via oxidative phosphorylation could occur at Δψ = −50 mV, but the baseline ATP efflux rate for Δψ ≥ −95 mV reported by Chinopoulos et al.^18^ shows that oxidative phosphorylation ceases when Δψ is above −95 mV.

### Δ*G*, Δ*S*, and Δ*H* for Forming the TELP Surface Layer

In our discussion above, we introduced Lee’s thermodynamic parameter Δμ_H+,P,L_ ≡ 2.3*RT*log(), which covered proton transport from the P, bulk phase to the P surface-TELP phase; this parameter is positive for [H^+^]_P,L_ > 0. Lee referred to this as Δ*G*_L_, but because his reported Δ*G*_L_ values are negative,^2^ it is clear that he changed the transport direction: Lee’s negative Δ*G*_L_ is for spontaneous proton transport from the P surface-TELP phase (high [H^+^]_P,L_) to the P, bulk phase (low [H^+^]_P,bulk_).

As with any other chemical process, we can break up free energy into enthalpy and entropy (eq 6):
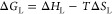
6

In a normal concentration cell, only solute concentration differs between the two phases; solvation is identical. Thus, we can approximate Δ*H*_L_ ≈ 0, and
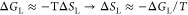
7

So for the “L” reaction, proton diffusion from the P surface to bulk phase (high to low concentration), Δ*S*_L_ will be positive and can be calculated from the equation
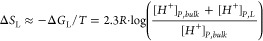
8

Unfortunately, Lee again reversed his signs here, writing Δ*G*_L_ ≈ *T*Δ*S*_L_ and calculating negative values for Δ*S*_L_. He wrote,^2^ “This study now, for the first time, numerically shows that transmembrane electrostatic proton localization (Type-B process) represents a negative entropy event with a local protonic entropy change (Δ*S*_L_) in a range from −95 to −110 J/K·mol.” Thus, using standard concentration cell thermodynamics, Lee has calculated an estimate of the entropy loss for proton transport from the low concentration P, bulk phase to the high concentration P, surface-TELP phase.

However, we must recall that the approximation sign in eq 8 stems from our assumption that Δ*H*_L_ ≈ 0, i.e., proton hydration is essentially identical in the surface and bulk phases. But recall that the structure and dielectric permittivity of the surface water layer differs dramatically from that in the bulk phase. Hence, Δ*H*_L_ ≈ 0 is a poor assumption. The parameter that Lee calculates as Δ*S*_L_, −Δ*G*_L_/T, is actually not a purely entropic term; rather, we can derive from eq 6 that it is actually Δ*S*_L_ – Δ*H*_L_/*T* (eq 9):
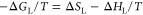
9

Given the high dielectric permittivity of bulk phase water (ε ≈ 80) relative to membrane surface water (ε ≈ 10),^25,27,38,42^ one would expect proton diffusion from bulk to surface to be endothermic. Thus, considering eq 9, the −Δ*G*_L_/*T* values tabulated by Lee are most likely more negative than the actual Δ*S*_L_ for the proton diffusion process.

### Redox-Attainable P Surface pH Values

Localized chemiosmosis for spontaneous proton flow through F_1_F_0_ to drive ATP synthesis can be described by eq 10, which is a modification of Mitchell’s original bulk phase equation:
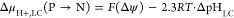
10Here, Δψ ≡ ψ_*N*_ – ψ_*P*_ is negative-inside, and ΔpH_LC_, defined as pH_N,surface_ – pH_P,surface_, is generally positive. Equation 10 can be rearranged to give
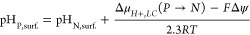
11

Assuming that Δμ_H+,LC_(*P* → *N*) is equivalent to that available from the redox energy input = −4.6 kcal/mol H^+^, and using pH_N_ = 7.41 (adjacent to the F_1_ complex) as reported by Rieger et al.,^15^ and *T* = 37 °C, eq 11 becomes
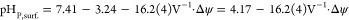
12

Using eq 12, we can calculate the minimum pH at the P surface that is attainable from redox energy input as a function of the Δψ maintained. These minimum attainable pH_P,surf_ values range from 7.1 for high-potential mitochondria (−180 mV) to 6.2 for low-potential mitochondria (−123 mV, see the Supporting Information, section IV). In agreement with these calculated pH minima, Cherepanov et al.^42^ predicted that attaining pH_P,surf._ below 6–6.5 would be highly unlikely (A. Mulkidjanian, person communication).

### Type-B Processes and the Evidence for Membrane Asymmetry

Lee has proposed that oxidative phosphorylation violates the second law in two different ways: B1, in which redox energy input is exceeded by the requisite ATP synthesis energy output; and B2, in which redox energy input is exceeded by the energy in the TELP surface-localized proton gradient. We showed in the first section above that Lee’s B1 conclusion is based on ATP synthesis energy calculations that are too high due to two different errors. Correcting these errors shows that there is no B1-type violation of the second law: Redox energy supplies 4.6 kcal/mol H^+^, and ATP synthesis requires 4.6 kcal/mol H^+^ in the matrix or 4.2 kcal/mol H^+^ in the cytoplasm.

On the other hand, Lee’s TELP-predicted gradients (pH_P,L_ ≈ 2) do present a B2-type violation of the second law, as long as one accepts his thermodynamically untenable ion exchange equilibrium constants (Table 1). As we mentioned above, Lee has argued in his recent papers that oxidative phosphorylation is an isothermal engine that can use thermal motion to do work, i.e., synthesize ATP: The establishment of the TELP P-surface localized proton layer is a type-B process that, due to structural and topological asymmetries in the bioenergetic membrane, is allowed to violate the second law.^1−4^

The literature on Type-B processes is an interesting subfield of physics and chemistry. Its critics claim that the second law of thermodynamics is a universal law, and experiments that demonstrate Type-B processes that seem to violate the law are either poorly designed or poorly carried out.^43^ On the other hand, supporters claim that their results are being censored from the peer-reviewed literature due to a superficial bias in favor of the universality of the second law, even though physicists in the 19th century already understood that the second law is not in fact universal.^44,45^ This is a fascinating controversy, but its details lie well beyond the scope of this paper. Here, we wish to examine the evidence in support of Lee’s contention that bioenergetic membrane asymmetry supports a type-B process.

Lee has proposed two distinct types of type-B supportive membrane asymmetries: lateral and longitudinal. Lateral asymmetry in the plane of the mitochondrial inner membrane has been strongly supported in the literature for 20 years or more. Three distinct regions of the mitochondrial inner membrane have been identified: highly curved cristae rims, cristae flat membranes, and flat inner boundary membranes (IBM, adjacent to the intermembrane space and across from the mitochondrial outer membrane). These regions feature distinct protein composition, Δψ, and ΔpH, which are summarized in Table 2.

**Table 2:** Examples of Inhomogeneity in the Mitochondrial Inner Membrane and Cristae

	**Cristae rims**	**Cristae flats**	**IBM**	**ref.**
enriched proteins	F_1_F_0_ dimers (proton sinks)	Complexes I, III, and IV (Proton sources)		(46, 47)
Δψ		–165 ± 10 mV	–155 ± 8 mV	(48)
pH_P_^a^	7.28 ± 0.24	6.88 ± 0.15		(15)
		6.88 ± 0.09		(49)
	7.20 ± 0.10	7.13 ± 0.17	6.85 ± 0.18	(17)
pH_N_^a^	7.41 ± 0.20	7.73 ± 0.24		(15)
		7.78 ± 0.11		(49)

a These values are measured with proton-sensitive GPF proteins fused to mitochondrial inner membrane proteins (Complexes IV and V). Although the GFP proton inlet sites are close to the membrane surface, it has been argued^28,22^ that they are not close enough to the headgroup/fatty acid interface to sample the surface layer pH.

The proton sources (redox-driven proton pumps, complexes I, III, and IV) are found primarily in the flat cristae membranes, whereas the proton sinks (dimeric F_1_F_0_ ATP synthases) are found primarily at the highly curved cristae rims.^47^ On the P side, protons are enriched at the cristae flat membranes and at the IBM compared to the cristae rims (ΔpH ≈ 0.35); on the N side, the difference is reversed, with proton enrichment at the cristae rims (ΔpH ≈ −0.35). Interestingly, this leads to a distinct difference in ΔpH, which is quite small at the cristae rims (≈ 0.13, ref (15)), and more substantial along the remainder of the cristae membrane (ΔpH ≈ 0.9). This makes sense when one considers that the F_1_F_0_ ATP synthase at the cristae rims consumes protons at the P surface and releases them on the N side.

Back in 2008, Strauss et al. reported^50^ that ribbons of F_1_F_0_ dimers helped to form cristae rims by “enforcing a strong local curvature on the membrane”. This curvature in turn caused an increase in charge density: Using Finite Element Modeling based on the Poisson equation to simulate electric field strength, they reported that for a crista axial ratio of 10:1, “the charge density on the curved membrane surface is up to 3.5 times higher than on planar membranes at the same constant membrane potential”. This would lead to proton enrichment at the crista rims, with pH_flat_ – pH_rim_ ≈ 0.5. Strauss et al. concluded that the purpose of mitochondrial cristae is not only to increase the surface area of inner membrane available to host oxidative phosphorylation complexes but also to focus protons at the F_0_ proton inlet, increasing ΔpH and the driving force and rate of ATP synthesis.^50^

Over a decade later, Lee published a paper^23^ reaching similar conclusions.^g^ Using an unpublished set of equations^51^ (from an undergraduate physics problem set) to calculate surface charge density along ellipsoid and disk surfaces, Lee calculated that a 10:1 crista axial ratio would yield proton enrichment at the crista rim of 1 pH unit. He did not explain the discrepancy that his result was twice that of Strauss et al. Nevertheless, the evidence supporting lateral asymmetry along the mitochondrial inner membrane and crista membrane seems strong.

**Figure 6:**
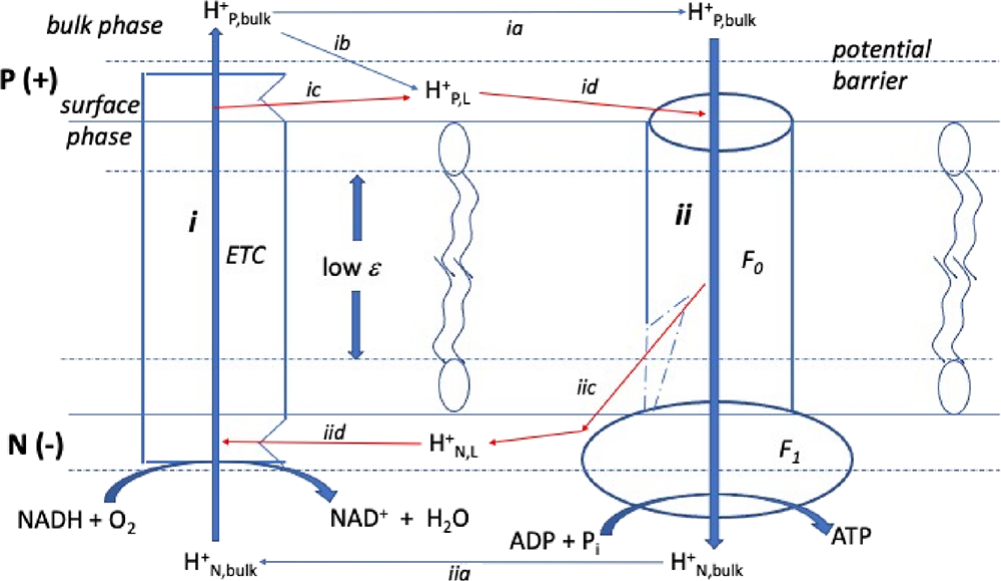
Longitudinal asymmetry in the mitochondrial inner membrane. Mitchellian bulk-to-bulk proton transport is symmetric: paths i → ia → ii → iia; Lee H^+^ transport is longitudinally asymmetric: i → ib → id → ii → iia; most recent structural results suggest that H^+^ transport is symmetric, along the surface: i → ic → id → ii → iic → iid.

Such is not the case for Lee’s contention of longitudinal asymmetry perpendicular to the plane of the membrane. Based on a schematic representation (essentially a cartoon drawing) published by Dudkina et al.^52^ in 2020, Lee concluded^23^ that the redox-driven proton pumps (complexes I, III, and IV) release protons into the bulk aqueous phase of the cristae lumen (P side, Figure 6), whereas the proton inlet in the F_0_ complex of the ATP synthase is right at the surface of the crista rim membrane. This leads to a “longitudinal” asymmetry on the P side between proton release into the bulk phase (path *i* on the left side of Figure 6) vs uptake from the surface layer (*id* in Figure 6). According to Lee, this longitudinal asymmetry figures prominently in the ability of surface proton gradients to violate the second law of thermodynamics (type-B process).

The problem is that this conclusion is based on a cartoon drawing in which proton release and uptake are depicted in the original paper simply by arrows: The actual sites of proton release are not identified; hence, one cannot conclude from this simple cartoon that the pumps release protons on the P-side into the bulk phase as opposed to the membrane surface. Since Dudkina’s 2010 paper, much better structural information on Complex I (NADH-UQ oxidoreductase) has been published.^53−55^ Figures in these papers make it clear that although the membrane arm of Complex I does protrude a few nm from the P surface of the crista membrane, the actual proton release sites lie almost flush with the membrane surface.

Similarly, although Complex III (UQ-cyt c oxidoreductase) protrudes tens of nanometers from the P surface, the Q_o/P_ ubiquinone binding site is leveled with the edge of the low dielectric interior of the membrane, near the headgroup region. Because Complex III utilizes a Q cycle mechanism of proton pumping, protons are released on the P side when UQH_2_ is oxidized at the Q_o/P_ site. Although the proton pathway leading from this site to the exterior of the protein is not yet fully characterized, extended H-bonded water chains have been found connecting each UQ binding site to the nearest membrane surface water layer (Jiapeng Zhu, personal communication). Thus, protons are likely to be released into the surface layer, not the bulk phase.

Finally, for Complex IV (cytochrome c oxidase), recent structural results have been reported for the three paths delineated for transmembrane proton pumping: the H-, D-, and K-channels.^56−59^ Although the protein surface of Complex IV extends several nanometers beyond both of the membrane surfaces, the proton inlet and outlet sites are generally within 0.1–0.5 nm of the lipid headgroup surface (i.e., within 1.2–1.6 nm of the low dielectric fatty acid/headgroup interface; see Supporting Information, section VII). Thus, they lie within the two water monolayers adjacent to the membrane surface.

In summary, Lee provided no evidence beyond a simple 2010 cartoon figure to support his contention of longitudinal asymmetry in proton source/sink machinery on the P side of the mitochondrial crista membrane. Recent high-resolution structural results suggest that such longitudinal asymmetry does not in fact exist: Pumped protons are most likely released into the same P side surface phase from which they are consumed by the F_1_F_0_ ATP synthase.^60,61^ They are also taken up from the same N side surface phase into which they are released by the F_1_F_0_ ATP synthase. We conclude from these recent structural results for Complexes I, III, IV, and V that proton flux is longitudinally symmetric, occurring along both membrane surfaces, as depicted by the red pathway arrows in Figure 6: *i → ic → id → ii → iic → iid*.

## Conclusions

- Redox energy input to oxidative phosphorylation equals or exceeds ATP synthesis energy output; hence, there is no violation of the second law of thermodynamics. For all mitochondria, from high potential (−180 mV) to low potential (−123 mV), the minimum pH_P,surface_ attainable from redox energy input (pH 7.1 or 6.2, respectively) is sufficient to support ATP synthesis.
- Given the physical and structural differences between bulk and surface water, in order to obtain the thermodynamic activity of surface protons, [H^+^]_surface_ must be multiplied by its activity coefficient, γ(H^+^)_surface_, which is currently unknown but is probably significantly different from 1.
- Lee’s TELP equations include several errors, including *C*/*A* = 1.32 vs 0.9 μF/cm^2^, surface layer thickness *l* = 1.0 vs 1.5 nm, and most significantly, ion exchange *K*_eq_ values ≈ 10^–8^ vs 10^–3^. Thus, his predicted total pmf values are likely to be much too high.
- Experimental corroboration of TELP predictions is lacking:
  - Reported pH_P,surface_ values range from 4.5 to 6.5; none are close to 2.
  - The dependence of alkaliphilic bacterial cell doubling rate on pH titrates with a p*K*_a_ = 8.51 ± 0.03; this sigmoidal decrease with pH differs significantly from the pH dependence of the TELP-predicted total pmf.
  - The linear decline of mitochondrial ATP efflux rate (and ATP synthesis) with Δψ, to a steady low baseline value at ≥ −95 mV, cannot be explained by dependence on the TELP-predicted total pmf; this latter value exceeds that required for ATP synthesis even at −50 mV.
- Lee’s calculation of Δ*S*_L_, a “novel” negative entropy event, contains several sign errors; more importantly, the value he calculates is actually −Δ*G*_L_/*T* (= Δ*S*_L_ – Δ*H*_L_/*T*), which is more negative than Δ*S*_L_ because Δ*H*_L_ is positive.
- Recent structural advances show that the longitudinal asymmetry claimed by Lee probably does not exist: Proton pumps most likely deliver protons into the same P-side surface layer from which they are drawn by the F_1_F_0_ ATP synthase.
- There is no evidence that oxidative phosphorylation violates the second law of thermodynamics. Sufficient energy for ATP synthesis can be supplied by an enhanced surface ΔpH, i.e., the difference pH_P,surface_ < pH_P,bulk_ envisioned by standard localized chemiosmosis, which posits a lack of bulk/surface proton equilibration due to a potential barrier between the two phases.^22,24^

## Supporting Information Available

Section I: Lee’s TELP model: implications of the first and second laws of thermodynamics; Section II: energy supplied by redox reactions is enough to drive ATP synthesis: |Δ*G*_redox_| ≥ Δ*G*_p_; Section III: capacitance cannot be used to calculate surface free energy or pmf; Section IV: meta-analysis (1969–2021) of mitochondrial transmembrane potentials; Section V: literature values for biological membrane-specific capacitance; Section VI: experimental measurements of pH at the membrane surface; Section VII: proton uptake and release sites in Complex IV (cytochrome c oxidase) (PDF)

## Footnotes

a Thermodynamic efficiencies (100% × Δ*G*_input_/|Δ*G*_output_|) are calculated here as 100% × Δ*G*_ATPsynth_/|Δ*G*_redox_|. These are the maximum theoretical thermodynamic efficiencies, in that we are ignoring here all detracting processes like proton leak, slip, uncoupling, variable stoichiometry, and other “mechanical” limitations of the enzymes involved.

b Lee reported these values as pmf = 228 mV and 254 mV, respectively, on p. 8 of ref (3).

c For a discussion of potential first law violations, see Section I of the Supporting Information.

d Diffusion coefficients,^31^ in nm^2^/ns, are 9.3 for H^+^, 5.3 for OH^–^, 2.0 for K^+^ and Cl^–^, 1.3 for Na^+^, 1.2 for HCO_3_^–^. Thus, H^+^ diffuses ≈5 to 8-fold faster than K^+^, Na^+^, Cl^–^, and HCO_3_^–^. Is this difference really enough to prioritize protons as the charge carrier in the bioenergetic “capacitor”?

e We have shown^22^ that the energy of electrostatic attraction is actually insufficient to hold H^+^/OH^–^ ion pairs at the surface. Over time, entropy drives spontaneous release into the bulk phase, dismantling the protonic capacitor.

f These values have been shown to be thermodynamically untenable^39^ and are likely to be orders of magnitude too low.

g However, one of Lee’s conclusions, “the formation of cristae creates more mitochondrial inner membrane surface area and thus more protonic capacitance for transmembrane-electrostatically localized proton energy storage”, is bioenergetically irrelevant. Lee’s TELP equations show that [H^+^]_L_ depends not on *C*, but on *C*/*A*, which is an area-normalized parameter and thus remains the same with and without cristae. Although the number of surface protons increases with cristae, so does the volume of the surface layer, leaving [H^+^]_L_, and thus the pmf driving force, unchanged.
