# Supplementary material for: Oxidative Phosphorylation Does Not Violate the Second Law of Thermodynamics
Source: J Phys Chem B. 2024 Aug 21;128(35):8448–58. doi: 10.1021/acs.jpcb.4c03047 (PMC11382260; doi:10.1021/acs.jpcb.4c03047)
Supplement: Supplementary file 1 — jp4c03047_si_001.pdf [file jp4c03047_si_001.pdf]

**Supporting Information** for

# *Oxidative Phosphorylation Does Not Violate the Second Law of Thermodynamics*

Todd P. Silverstein

Department of Chemistry (emeritus), Willamette University, Salem, OR 97301 (USA)

## I. *Lee's TELP model: implications of the first and second laws of thermodynamics*

Lee has written that the energy stored in his predicted TELP protonic capacitor violates the second law of thermodynamics, but not the first law. Recall that according to Mitchell's Chemiosmotic Theory, bioenergetic membranes contain enzymes that catalyze two fundamental processes: redox-driven proton pumping to set up a high-energy proton gradient, and proton-influx-driven ATP synthesis. So, the proton gradient is the high-energy intermediate established by spontaneous redox reactions, and drained by the ATP synthase. According to Lee, the redox free energy available in oxidative phosphorylation is 5.26 kcal/mol $H^+$, whereas 5.85 kcal/mol $H^+$ is required to drive ATP synthesis. Furthermore, according to Lee's model, the energy stored in the TELP protonic capacitor resulting from redox-driven proton pumping ranges from 9.5 kcal/mol $H^+$ (for $\Delta\psi$ = -81 mV) to 12.3 kcal/mol $H^+$ (for $\Delta\psi$ = -180 mV; see Table 1 in our accompanying paper). Let's approximate this as 11 kcal/mol $H^+$.

### *A. First law of thermodynamics: TELP system/redox surroundings*

This raises the question: If only 5 kcal/mol $H^+$ is available from redox reactions, how can the redox-driven proton gradient be energized to 11 kcal/mol $H^+$? Let us examine this question first from the perspective of the first law of thermodynamics (Equation S*1*):

Equation S1: $$\Delta U_{sys} = q_{sys} + w_{sys}$$

where $q_{sys}$ = energy transferred in the form of heat; if heat flows from the surroundings to the system $q_{sys}$ is positive, and for the reverse flow it is negative; $w_{sys}$ = energy transferred in the form of work, with sign conventions and energy flux directions as noted above; $\Delta U_{sys}$ is the change in total internal energy of the system.

If the universe is isolated, with no flow of energy in or out, then conservation of energy can be expressed as:

Equation S2: $\Delta U_{\text{universe}} = \Delta U_{\text{system}} + \Delta U_{\text{surroundings}} = 0 \Rightarrow \Delta U_{\text{system}} = -\Delta U_{\text{surroundings}}$

Thus, in order to employ the first (and second) law of thermodynamics, one must define a universe (system + surroundings) that is isolated. That is a problem in biochemistry, as almost all biochemical processes are connected to other biochemical processes. For example, the reduced substrate inputs to the redox reactions in oxidative phosphorylation (NADH and $FADH_2$) are produced mostly in the citric acid cycle, and the pyruvate input into the citric acid cycle is produced by glycolysis. Similarly, the high-energy proton gradient produced by the redox reactions is drained by the ATP synthase. To claim as Lee has done, that his TELP protonic capacitor model obeys the first law of thermodynamics, one must assume that the redox/TELP universe is isolated: reduced substrates do not enter, and energy neither enters nor leaves. Clearly the former assumption is false: Both NADH and $FADH_2$ enter the redox surroundings from glycolysis and the citric acid cycle; furthermore, we shall demonstrate below that the latter assumption is also false.

First, let us define the *system* as Lee's TELP protonic capacitor, and the *surroundings* as the redox reactions that drive proton pumping. With these definitions, we can write

Equation S3: $\Delta U_{\text{sys}} = q_{\text{TELP}} + w_{\text{TELP}}$ and $\Delta U_{\text{surr}} = q_{\text{redox}} + w_{\text{redox}}$

Next, since $\Delta G$ is the maximum amount of energy available to do non-expansion work at constant *T, P*, we can estimate $w_{\mathrm{TELP,max}} \approx 11$ kcal/mol $H^+$, and $w_{\mathrm{redox,max}} \approx -5$ kcal/mol $H^+$; $w_{\mathrm{redox}}$ is negative because the redox reactions (surroundings) are doing work on the TELP gradient (system). If one assumes that the surroundings are isolated, then the first law requires conservation of energy; combining Equations S2 and S3, we have

Equation S4: $q_{\mathrm{TELP,min}} + 11 \text{ kcal/mol } H^+ = -(q_{\mathrm{redox,min}} - 5 \text{ kcal/mol } H^+)$, and

Equation S5: $-q_{\mathrm{redox,min}} = q_{\mathrm{TELP,min}} + 6 \text{ kcal/mol } H^+$

Thus, the minimum amount of heat flowing from the surroundings (redox reactions) exceeds the minimum amount of heat flowing into the TELP protonic capacitor by 6 kcal/mol $H^+$. As we shall see below, this excess heat does not actually derive from the redox reactions (which emit very little heat) but rather from mitochondrial reactions that lie outside of our universe as defined above. Thus, we have demonstrated that the redox/TELP universe is not energetically isolated, and $\Delta U_{\mathrm{universe}}$ is therefore not required to be zero. Contrary to Lee's conclusion, the first law of thermodynamics can NOT be applied to this simple redox/TELP universe.

*B. Expanding the system to include ATP synthesis*

To understand the conundrum of the 6 kcal/mol $H^+$ of "excess" heat, it is instructive to expand our system to include the subsequent bioenergetic reaction, proton-driven ATP synthesis. As noted above, according to Lee, $\Delta G_{\mathrm{redox}} \approx -5$ kcal/mol $H^+$, $\Delta G_{\mathrm{TELP}} \approx +11$ kcal/mol $H^+$. Furthermore, proton-driven ATP synthesis involves proton influx down its gradient yielding -11 kcal/mol $H^+$ and concomitant ATP synthesis requiring +6

kcal/mol $H^+$ (according to Lee), so $\Delta G_{ATPsynth} \approx$ -5 kcal/mol $H^+$. The net $\Delta G$ of these three processes is then +1 kcal/mol $H^+$ (-5 + 11 – 5). Although on a per proton basis the positive $\Delta G_{net}$ suggests nonspontaneity, we must consider the stoichiometry of these processes: NADH oxidation is coupled to the pumping of 10 protons, which in turn energizes the synthesis of 3.75 ATP (= 10 H+/NADH ÷ 2.67 H+/ATP[*]). Thus, per NADH, $\Delta G_{net}$ of these three processes = -28 kcal/mol NADH (= 10·(-5) + 10·(11) + 10·(–11) + 3.75·(6)). Other non-bioenergetic processes carried out in the mitochondrion are all spontaneous as well, e.g., -13.5 kcal/mol NADH for glycolysis, -11.5 kcal/mol NADH for citric acid cycle[1], and would make $\Delta G_{mito}$ even more negative.

In summary, when considering only the first two bioenergetic components, with redox-driven proton pumping defined as the surroundings, and Lee's protonic capacitor as the system, there is a heat gap of 6 kcal/mol $H^+$, and reduced substrates flow in from outside of the surroundings. We can therefore conclude that this defined universe is not isolated, hence neither the first law nor the second law can be applied to this redox/TELP universe (without further accounting for energy and matter transfer into and out of this universe). On the other hand, when we broaden the system to include the next bioenergetic reaction, proton-driven ATP synthesis, there is no energy gap, and no thermodynamic problem.

[*] This coupling factor of 2.67 H+/ATP is for ATP synthesized inside the mitochondrial matrix. For ATP exported to the cytoplasm, the coupling factor is 3.67 H+/ATP, which would make $\Delta G_{net}$ even more negative: -34 kcal/mol NADH.

*II. ATP synthesis energy required ≤ energy supplied by redox reactions*

*A. Redox free energy*

For the oxidation of NADH ($E^{o\prime}$ = -0.315 V) by $O_2$ ($E^{o\prime}$ = +0.816 V) via the electron transfer chain (ETC) coupling sites, $\Delta E^{o\prime}$ = +1.131 V, so $\Delta G^{o\prime}$ = -52.14 kcal/mol NADH (= $-2 \cdot F \cdot \Delta E^{o\prime}$). Because NADH oxidation involves all three coupling sites and pumps 10 protons from the N to the P side, $\Delta\mu_{H+}$ = -52.1 kcal/mol NADH ÷ 10 $H^+$ pumped/NADH = -5.21 kcal/mol $H^+$. This value is calculated for the biochemical standard state, at which all reactant and product concentrations (except for $H^+$) are 1 M, but that is decidedly not the case in functioning bioenergetic systems. Using typical steady state mitochondrial concentrations of aqueous NADH, $NAD^+$, and $O_2$, we reported $\Delta\mu_{H+}$ = -44.5 kcal/mol NADH ÷ 10 $H^+$ pumped/NADH = **-4.45 kcal/mol $H^+$.**[1] Similar calculations for $FADH_2$ oxidation ($E^{o\prime}$ = +0.05 V) give $\Delta G^{o\prime}$ = -35.3 kcal/mol $FADH_2$ and $\Delta G$ = -28.1 kcal/mol.[1] $FADH_2$ is oxidized by only two coupling sites (Complexes III and IV) along with the pumping of 6 protons, so the maximum $\Delta\mu_{H+,FADH2}$ would be **-4.68 kcal/mol $H^+$**. Thus, a reasonable estimate of the attainable chemical potential in the redox-driven proton gradient is **$\Delta\mu_{H+,redox} \approx$ -4.6 kcal/mol $H^+$.**

B. *ATP synthesis free energy*

For the synthesis of ATP, Lee used[2,3] a value reported by Cockrell et al in 1966, $\Delta G_p$ = +15.6 kcal/mol ATP.[4] However, Cockrell et al. did not measure $\Delta G_p$ in typical respiring mitochondria. To quote the original article: "The experimental conditions employed… have been deliberately chosen as requiring a higher $\Delta F'$ [i.e., $\Delta G_p$] for ATP formation than the assumed conditions of Mitchell."[4] Thus, Cockrell et al set up an artificially high $\Delta G_p$ of 15.6 kcal/mol ATP, compared to the value employed by Mitchell (9.6 kcal/mol).

$\Delta G_p$ has been measured extensively in the cytoplasm, and somewhat less extensively in the mitochondrial matrix. To determine $\Delta G_p$, one must know $\Delta G_p^{o\prime}$, the [ATP]/[ADP] ratio, and [$P_i$]. We start with $\Delta G_p^{o\prime}$ = +8.6 kcal/mol ATP at $I$ = 0.2 M, 1 mM $Mg^{2+}$, 25 °C, and pH 7.[5] Rieger et al[6] reported a pH of 7.41 at the $F_1$ (site of ATP synthesis), and using the slope of 1.297 kcal/mol/pH unit from ref. [5], we calculate $\Delta G_p^{o\prime}$ = +9.1(3) kcal/mol ATP at pH 7.41. Additionally, given $\Delta S_p$ = +18.8 cal/K/mol ATP,[7,8], a 12°C increase from 25 to 37 °C would lower $\Delta G_p^{o\prime}$ by 0.225 kcal/mol, to **8.9 kcal/mol ATP**. This matches values of $\Delta G_p^{o\prime}$ reported for chloroplasts[9] and yeast[10] (8.6 ± 0.7 kcal/mol), thermophilic *Bacillus* PS3[11] (8.85 ± 0.24 kcal/mol), and *E. coli*[12] (9.1 ± 0.7 kcal/mol).

In section C below, we report the collected results of our literature meta-analysis, listing reported values of [ATP]/[ADP] ratio, [$P_i$], $Q_p$ = [ATP]/[ADP]/[$P_i$], and $\Delta G_p = \Delta G_p^{o\prime} + RT\ln Q_p$, in both the cytoplasm and the mitochondrial matrix. We find **$\Delta G_p$ = 12.3 ± 0.7 kcal/mol in the matrix,** and **15.4 ± 1.1 kcal/mol in the cytoplasm**. (It is interesting how close this latter value is to that employed by Cockrell et al in their 1966 experiments.)

Mitochondrial $F_1F_o$ synthesizes ATP in the matrix. The vertebrate mitochondrial $F_o$ complex has 8 proton-binding *c* subunits, while $F_1$ has 3 ATP synthesizing $\beta$ subunits, thus the $H^+$/ATP coupling factor = 8 $H^+$/3 ATP = 2.67 $H^+$/ATP. The $\Delta\mu_{H+}$ required to drive ATP synthesis by $F_1$ in the matrix is thus 12.3 kcal/mol ATP ÷ 2.67 $H^+$/ATP = **4.6 kcal/mol $H^+$.** Interestingly, this is the exact value of proton electrochemical gradient free energy available from the redox reactions (Section A above).

Intact mitochondria must export ATP to, and import ADP from, the cytoplasm (via the ATP/ADP exchange antiporter), and import phosphate from the cytoplasm. The net effect of these transport processes is to require the import of 1 additional proton per ATP; hence the operative $H^+$/ATP coupling factor in intact state 3 mitochondria = 11/3 = 3.67 $H^+$/ATP. Thus, to drive ATP synthesis and export to the cytoplasm, $\Delta\mu_{H+}$ would have to be at least 15.4 kcal/mol ATP ÷ 3.67 $H^+$/ATP = **-4.2 kcal/mol $H^+$**; the **-4.45 kcal/mol $H^+$** available from NADH oxidation (section A above) more than suffices.

### C. *Meta-analysis of ATP/ADP ratio and ADP phosphorylation free energy ($\Delta G_p$)*

1. *Measured in cytosol (in vivo) or external medium for isolated mitochondria*

| **Phase**[a] | **[ATP]/ [ADP]** | **[$P_i$], mM** | **$Q_p$**[b] | **$\Delta G_p$**[c] **(kcal/ mol)** | **Exptl. mitoch. system** | **Exptl. method** | **Ref.** |
|---|---|---|---|---|---|---|---|
| Ext. | | | 616,600 | 17.1 | Muscle, rat | Equilibrated enzymes | [13] |
| Ext. | | | 389,000 | 16.8 | Muscle, human | Equilibrated enzymes | [13] |
| Ext. | | | 339,000 | 16.75 | Heart, rat | $^{31}$P, perfused organ | [13] |
| Ext. | | | 190,550 | 16.4 | Muscle, human | $^{31}$P, perfused organ | [13] |
| Ext. | | | 204,200 | 16.4 | Muscle, human | $^{31}$P, perfused organ | [13] |
| Ext. | | | 164,000 ±110,000 | 16.2 ± 0.5 | Isolated mitoch | unspecified | [14] |
| Ext. | | | 129,000 | 16.1 | Heart, rat | Equilibrated enzymes | [13] |
| Ext. | | | 93,300 | 16.0 | Brain, rat | Equilibrated enzymes | [13] |
| Cyt. | 534 | 7.0 | 76,000 | 15.8 | Muscle & brain, rat | $^{31}$P, whole animal | [15,16] |
| Ext. | 111 ± 25 | 1.73 ± 0.4 | 64,100 ±21,000 | 15.72 ± 0.28 | Brain, dog | $^{31}$P, whole animal | [17] |
| Ext. | | | 55,000 | 15.6 | Heart, rat | $^{31}$P, perfused organ | [13] |

| | | | | | | | |
|---|---|---|---|---|---|---|---|
| Ext. | | | 40,700 | 15.4 | Brain, gerbil | $^{31}$P, perfused organ | 13 |
| Ext. | 14 ± 5 | 0.7 ± 0.5 | 6400 ± 3000 | 14.2 ± 0.5 | Isolated mitoch. | Scintillation counting | 18 |
| Ext. | 29 | 0.48 | 60,400 | 15.4 | Isolated mitoch. | Scintillation counting | 19 |
| Cyt. | 80.9 ± 2.7 | 2.72 ± 0.06 | 29,800 ±1,200 | 15.25 ± 0.10 | Brain, rat | Equilibrated enzymes | 20 |
| Cyt. | 218 ± 6 | 8.0 ± 0.4 | 27,200 ±1,700 | 15.19 ± 0.11 | Muscle, rate | Equilibrated enzymes | 20 |
| Ext. | | | 20,600 ± 13,000 | 14.9 ± 0.4 | Isolated mitoch | unspecified | 14 |
| Cyt. | 73.5 ± 5 | 4.76 ± 0.16 | 15,440 ±1,200 | 14.84 ± 0.11 | Liver, rat | Equilibrated enzymes | 20 |
| Cyt. | 27.6 ± 5 | 2.0 ± 0.1 | 13,800 ±2,600 | 14.77 ± 0.23 | Liver, rat | Column chromatogr. | 21 |
| Cyt. | 39.2 | 4.8[d] | 8167 | 14.35 | Heart, rat (rest) | Computational model | 22 |
| Cyt. | 28.0 | 3.5[d] | 8007 | 14.34 | Heart, rat (+$O_2$) | Computational model | 22 |
| Cyt. | 19.0 | 2.7[d] | 7017 | 14.26 | Heart, rat (Tbl3) | Computational model | 22 |
| Cyt. | 52.5 | 8.1[d] | 6485 | 14.21 | Heart, rat (+gluc) | Computational model | 22 |
| Cyt. | 9.1 ± 0.6 | 1.65 ± 0.12 | 5500 ± 500 | 14.21 ± 0.12 | rbc, human | Equilibrated enzymes | 20 |
| Cyt. | 8.8 ± 0.4 | 6.6 ± 0.3 | **1330** ± 100 | 13.33 ± 0.20 | Liver, rat | Column chromatogr. | 23 |
| Cyt. | 6.5 ± 0.1[e] | 16.7[e] | **390** | 12.6[e] | Heart, rat | Unspecified[e] | 24 |
| Ext. | 435 | | | | Isolated mitoch. | Kinetic comp. model | 25 |
| Ext. | 50 ± 8 | | | | Isolated mitoch. | Unspecified | 26 |
| Ext. | 53 ± 14 | | | | Isolated mitoch. | Equilibrated enzymes | 27 |
| Ext. | 23 ± 6 | | | | Isolated mitoch. | Equilibrated enzymes | 27 |
| Cyt. | 10.1 ± 3 | | | | Wheat protoplst | Equilibrated enzymes | 28 |
| Cyt. | 15.7 ± 2.1 | | | | Sycamore cells | $^{31}$P NMR | 29 |

[a] int.: mitochondrial matrix (internal); ext.: cytoplasm (in vivo), external medium (in vitro)
[b] reaction quotient for ATP synthesis: $Q_p$ = [ATP]/[ADP]/[$P_i$]
[c] $\Delta G_p = \Delta G^{o\prime} + RT\ln Q_p$; $\Delta G^{o\prime}$ = 8.9 kcal/mol at pH 7.4 (8.6 kcal/mol at pH 7.0)[5,30]
[d] $[P_i]_{cyt}$ calculated from ref. [16]
[e] Steenbergen et al did not specify how they measured nucleotide and phosphate concentrations. From their relatively high [$P_i$] and low [ATP]/[ADP] it seems likely that they measured total concentration of $P_i$ and ADP, instead of the free concentration: About 2/3 of cytoplasmic ADP is known to bind to actin,[22,31,32,33], whereas phosphate readily complexes with $Ca^{2+}$.[34,35,36,37] The Steenbergen et al value of $\Delta G_p$ = 12.6 kcal/mol is thus likely to be too low; furthermore, as judged by the Q-test, both their value of $\Delta G_p$ = 12.6 kcal/mol and [$P_i$] = 16.7 mM are statistical outliers (Q-test value > $Q_{90\%}$).

2. *Measured in mitochondrial matrix (interior phase):*

| **Int.** or **ext.**[a] | **[ATP]/ [ADP]** | **[$P_i$], mM** | **$Q_p$[b]** | **$\Delta G_p$[c] (kcal/ mol)** | **Exptl. mitoch. system** | **Exptl. method** | **Ref.** |
|---|---|---|---|---|---|---|---|
| Matrix | 2.4 ± 1.5 | 2.4 | 1010 ± 200 | 13.2 ± 0.4 | Isolated mitoch. | Equilibrated enzymes | 16 |
| Matrix | 3.9 | 3.5 | 1110 | 12.9 | Isolated mitoch. | Scintillation counting | 19 |
| Matrix | 1.90 ± 0.15 | 3.47 ± 0.15 | 542 ± 50 | 12.78 ± 0.21 | Liver, rat | Column chromatogr. | 23 |
| Matrix | 4.3 ± 0.3 | 17.3 ± 0.4 | 249 ± 21 | 12.30 ± 0.21 | Liver, rat | Column chromatogr. | 21 |
| matrix | 2.14 ± 0.4 | 37 ± 1[f] | 58 ± 10 | 11.40 ± 0.23 | Isolated mitoch. | Kinetic com-put’l model | 25 |
| Matrix | 4.8 ± 0.6 | | | | Isolated mitoch. | Unspecified | 26 |
| matrix | 4.1 | | | | Heart, rat (rest) | Computational model | 22 |
| matrix | 4.0 | | | | Heart, rat (+$O_2$) | Computational model | 22 |
| matrix | 3.9 ± 1.5 | | | | barley protoplst | Equilibrated enzymes | 38 |
| matrix | 3.3 | | | | Heart, rat (+gluc) | Computational model | 22 |
| Matrix | 3.3 ± 0.5 | | | | Isolated mitoch. | Equilibrated enzymes | 27 |
| Matrix | 3.2 ± 0.6 | | | | Isolated mitoch. | Equilibrated enzymes | 27 |

| | | | | | | | |
|---|---|---|---|---|---|---|---|
| Matrix | 2.6 ± 0.6 | | | | Wheat protoplst | Equilibrated enzymes | [28] |
| matrix | 2.4 | | | | Heart, rat (Tbl3) | Computational model | [22] |
| Matrix | 2.4 ± 0.4 | | | | Sycamore cells | $^{31}$P NMR | [29] |

[f] Phosphate concentration cited from ref. [39]; this concentration is 2 – 15x higher than recently reported values, perhaps because it includes both free and bound phosphate.

The most robust conclusion from comparing literature values for mitochondrial exterior vs. interior (matrix) phases is that $\Delta G_p$ for ATP synthesis is clearly lower in the interior matrix phase: 12.6 ± 0.7 kcal/mol (average ± standard deviation; range: 11.4 to 13.2 kcal/mol) vs. 15.4 ± 1.1 kcal/mol (range: 13.3 to 17.1 kcal/mol); the difference is statistically significant, with $P = 1.3(10^{-6})$. This difference of 2.8 kcal/mol (or 2.6 kcal/mol for the medians) is matched by the 2.5 ± 0.2 kcal/mol difference observed in the two literature reports that measured both values in the same experimental system[21],[19]; Klingenberg reported a difference ranging from 1.8 to 3.4 kcal/mol.[40]

Similarly, the [ATP]/[ADP] ratio is lower in the matrix: 3.2 ± 0.9 (range: 1.9 to 4.8) vs. 34 ± 140 (median ± standard deviation; range: 6.5 to 534). Although the range and standard deviation in the cytoplasm/exterior phase is quite large, the difference between the matrix vs. cytoplasmic [ATP]/[ADP] ratios is statistically significant, with $P = 0.026$. Klingenberg reported that the ratio is typically 5-fold lower in the matrix, and can be up to 20-fold lower.[40] He also reported that the phosphate concentration in the matrix is about 3-fold higher than that in the cytoplasm[40], and the average of reported values bears this out: 13 ± 15 mM (range: 2.4 to 17 mM) vs. 4 ± 4 mM (range: 0.5 to 8 mM). However, judging

by the large standard deviations, the difference is statistically suspect; this is borne out by the two-tailed *P*-value, 0.052 which is slightly above the cutoff value of 0.05.

Using matrix values of 3.2 ± 0.9 for the ATP/ADP ratio, 13 ± 15 mM for [$P_i$], and ΔG°' = 8.9 kcal/mol at pH 7.4, we find that $\Delta G_p$ for ATP synthesis is **12.3 ± 0.8 kcal/mol** in the matrix. This matches well with the five literature reports, which averaged **12.6 ± 0.7** kcal/mol, and the **median value of 12.8 kcal/mol**.

## *III. Capacitance cannot be used to calculate surface free energy or pmf*

The crux of Lee's TELP hypothesis is that in the presence of a transmembrane potential, excess protons and hydroxide anions, due to their enhanced diffusion, array themselves at opposing membrane surfaces the way that electrons and electron holes do on a pair of capacitor plate surfaces in an electronic circuit. According to the laws of electrostatics, the surface charge density (moles of charge per area) can be calculated from the voltage and the specific capacitance of the medium between the plates:

Equation S6: charge density (mol/area) = $(C/A) \cdot \Delta\psi / F$

where $C$ = capacitance, $A$ = plate area, $\Delta\psi$ = voltage, and $F$ = Faraday's constant.

Due to the delocalized conduction bands in metals, excess electrons in a metal behave as a continuous fluid (or gas) whose particles are minuscule. Because of this, in a charged capacitor, electrons are found only at the surface of the negative capacitor plate closest to the positive plate. Thus, there is no measurable volume associated with the electron layer on the capacitor plate surface, only an area.

Lee modified this equation by assuming a specific thickness, *l*, for the "plate" at the membrane surface; localized excess protons are found only within this distance *l* from the low dielectric membrane interior core. The idea that there is an abrupt distance cutoff separating the bulk aqueous phase from the localized surface layer has been challenged.[41] Nevertheless, selecting a specific value of *l* allowed Lee to divide the charge density by *l*, arriving at a concentration of excess charge within the localized

TELP layer, in units of moles per liter. After accounting for ion exchange between surface protons and bulk metal cations (e.g., $Na^+$, $K^+$), the molar concentration of excess protons within the localized TELP layer can be calculated. (However, the ion exchange equilibrium constants employed by Lee have been shown to be untenable.[42])

The electrochemical potential difference for a concentration cell where an ionic solute, $M^{z+}$, moves from phase 1, where its concentration is $[M^{z+}]_1$, to phase 2, where its concentration is $[M^{z+}]_2$, across a membrane that features a transmembrane potential of $\Delta\psi \equiv \psi_2 - \psi_1$, is given by Equation S7.

Equation S7: $$\Delta\mu_{M+}(1 \rightarrow 2) = F(\Delta\psi) + 2.3RT\log(\frac{a_{M_2^{z+}}}{a_{M_1^{z+}}})$$

where for solute $i$: $a_i = \gamma_i \cdot c_i$: $a$ = activity, $\gamma$ = activity coefficient.

The chemiosmotic equation for spontaneous proton transport from the P to the N phase is usually given by Equation S*8*:

Equation S8: $$\Delta\mu_{H+}(P \rightarrow N) = F(\Delta\psi) + 2.3RT\log(\frac{[H^+]_N}{[H^+]_P})$$

where $\Delta\psi \equiv \psi_N - \psi_P$ is negative-inside, and generally, $[H^+]_P > [H^+]_N$.

We must point out however that Equation S*8* is actually an approximation. The exact equation, derived from Equation S*7*, is

Equation S9: $\Delta\mu_{H+}(P \rightarrow N) = F(\Delta\psi) + 2.3RT\log(\frac{\gamma_{H_N^+} \cdot [H^+]_N}{\gamma_{H_P^+} \cdot [H^+]_P})$

Equation S9 only reduces to Equation S*8* if $\gamma_{H_N^+} = \gamma_{H_P^+}$. In the bulk aqueous phase, as long as $[H^+] < 0.01$ M (pH 2), we can in fact assume that $\gamma_{H_N^+} = \gamma_{H_P^+} \approx 1$. Thus, Mitchell's delocalized chemiosmotic equation for bulk to bulk proton transport is accurate:

Equation S10: $\Delta\mu_{H+}(P_{bulk} \rightarrow N_{bulk}) = F(\Delta\psi) + 2.3RT\log(\frac{[H^+]_{N,bulk}}{[H^+]_{P,bulk}})$

For localized chemiosmotic proton transport from surface to surface, the equation for electrochemical potential would be:

Equation S11: $\Delta\mu_{H+}(P_{surf} \rightarrow N_{surf}) = F(\Delta\psi) + 2.3RT\log(\frac{\gamma_{H_N^+} \cdot [H^+]_{N,surf}}{\gamma_{H_P^+} \cdot [H^+]_{P,surf}})$

By making a seemingly arbitrary assumption about the specific value of $l$, the localized surface layer thickness, Lee was able to convert protonic "capacitor" charge density to surface molar concentration, $[H^+]_{P,surf.}$. However, in his TELP pmf calculations, Lee used the following version of Equation S*11*:

Equation S12: $\Delta\mu_{H+}(P_{surf} \rightarrow N_{surf}) = F(\Delta\psi) + 2.3RT\log(\frac{[H^+]_{N,bulk}}{[H^+]_{P,surf}})$

In order to go from Equation S*11* to Equation S*12*, Lee made two implicit (and unwarranted) assumptions. First, he assumed $\gamma_{H_P^+} \approx 1$. There is a fair amount of evidence that the structure of water adjacent to the membrane surface is quite different from that in the bulk phase.[43,44,45,46,47] For one thing, the dielectric constant of surface water is much lower (≈ 10) than that in the bulk phase (≈ 80).[47,44,43,45] Thus one cannot assume that $\gamma_{H+}$ at the membrane surface is approximately one.

Second, in place of the log term numerator, $\gamma_{H_N^+} \cdot [H^+]_{N,\mathrm{surf}}$, Lee used $[H^+]_{N,bulk}$. In other words, he assumed that protons at the membrane's N surface are in a phase identical to the bulk phase, where the pH is approximately 7. This runs counter to the assumptions of both localized chemiosmosis, and also counter to Lee's own TELP hypothesis. According to TELP, the number of excess protons in the P side TELP layer is identical to the number of excess hydroxide anions in the N side TELP layer. Therefore, assuming that the water auto-protolysis equilibrium holds at the membrane surface as it does in bulk water, then if pH in the P side TELP surface layer is ≈ 2, then in the N side TELP surface layer pH must be ≈ 12. If, as seems likely, $K_w$ is lower in the low-dielectric surface phase, the problem is even worse. For example, assuming $K_w = 10^{-16}$, then pH ≈ 2 in the P-side TELP layer would imply pH ≈ 14 in the N-side layer. Therefore, Lee's assumption that $\gamma_{H_N^+} \cdot [H^+]_{N,\mathrm{surf}} \approx [H^+]_{N,bulk} \approx 10^{-7}$ M is untenable.

In summary, we have shown here that Lee's TELP pmf calculations using Equation S*12* are thermodynamically unsound.

*IV. Meta-analysis (1969-2021) of mitochondrial transmembrane potentials*

**Table S1**: Mitochondrial transmembrane potentials ($\Delta\psi$) in vivo (A) and in vitro (B).

*Table S1A: mitochondria **in vivo** – cell culture, perfused organ, live animal*

| $\Delta\psi$ (mV)[a] | source | method | Ref. |
|---|---|---|---|
| 118.2 ± 1.4 | Whole perfused heart[b1] | $[^3H]TPP^+$ distribution | 48 |
| 108.0 ± 1.5 | Whole perfused heart[b2] | $[^3H]TPP^+$ distribution | 48 |
| 100.8 ± 1.0 | Whole perfused heart[b3] | $[^3H]TPP^+$ distribution | 48 |
| 121.8 ± 1.5 | Whole perfused heart[b4] | $[^3H]TPP^+$ distribution | 48 |
| 110.4 ± 4.7 | Whole perfused heart[b5] | $[^3H]TPP^+$ distribution | 48 |
| 91 ± 11 | whole perfused heart | $[^{18}F]TPP^+$ distribution | 49 |
| 81 ± 13 | Live animal | $[^{18}F]TPP^+$ distribution | 49 |
| 117.6 ± 1.3 | Live animal | $[^{18}F]TPP^+$ distribution | 50 |
| 123 | Live animal[2] | $[^{18}F]TPP^+$ distribution | 51 |
| 125 | whole perfused heart | $[^3H]TPMP^+$ distribution | 52 |
| 105.3 ± 0.9 | fibroblast cell culture[c1] | $TMRM^+$ fluorescence | 53 |
| *81.3 ± 0.7* | neuroblastoma cell culture[c2] | $TMRM^+$ fluorescence | 53 |
| 116 | brown fat cell culture | $[^3H]TPP^+$ distribution | 54 |
| 154 ± 20 | liver cell culture | $[^3H]TPP^+$ distribution | 55 |
| 161 ± 3[m] | liver cell culture | $[^3H]TPP^+$ distribution | 56 |
| 148 ± 6 | cortical neuron synaptosomes | $[^3H]TPMP^+$ distribution | 57 |
| **139 ± 25** | cortical neuron cell culture[d1] | $TMRM^+$ fluorescence | 58 |
| **158 ± 21** | cortical neuron cell culture[d2] | $TMRM^+$ fluorescence | 58 |
| **166.8 ± 2.1** | Pancreatic islet cell culture[k] | $TMRM^+$ fluorescence | 59 |
| **178 ± 20** | Pancreatic islet cell culture[k1] | $TMRM^+$ fluorescence | 59 |
| **140 ± 3** | Pancreatic islet cell culture[k2] | $TMRM^+$ fluorescence | 59 |
| **151 ± 5** | Pancreatic islet cell culture[k3] | $TMRM^+$ fluorescence | 59 |
| **157 ± 8** | Pancreatic islet cell culture[k4] | $TMRM^+$ fluorescence | 59 |
| **163 ± 10** | Pancreatic islet cell culture[k5] | $TMRM^+$ fluorescence | 59 |
| **166 ± 10** | Pancreatic islet cell culture[k6] | $TMRM^+$ fluorescence | 59 |
| **141 ± 4** | Insuloma cell culture | $TMRM^+$ fluorescence | 59 |
| **172 ± 8** | Myoblast cell culture, H9c2 | $TMRM^+$ fluorescence | 60 |
| **183 ± 7** | Hepatoma cell culture, HepG2 | $TMRM^+$ fluorescence | 60 |
| **134 ± 12** | Fibroblast cell culture, BJ1 | $TMRM^+$ fluorescence | 60 |
| **165 ± 10** | HeLa cell culture[e1] | $TMRE^+$ fluorescence | 61 |
| **152 ± 8** | HeLa cell culture[e2] | $TMRE^+$ fluorescence | 61 |
| **150** | cerebellar neuron cell culture | $TMRM^+$ fluorescence | 62 |
| **155 ± 2** | Macrophage cell culture[f1] | Cyt $bc_1$ redox poise | 63 |
| **180 ± 3** | Macrophage cell culture[f2] | Cyt $bc_1$ redox poise | 63 |
| **150** | Human leukemia cell culture[g1] | Cyt $bc_1$ redox poise | 64 |

| | | | |
|---|---|---|---|
| **164** | Human leukemia cell culture[g2] | Cyt $bc_1$ redox poise | [64] |
| **178 ± 4** | Kidney 293 (wt) cell culture | Cyt $bc_1$ redox poise | [65] |
| **162 ± 2** | Kidney 293 (kd) cell culture | Cyt $bc_1$ redox poise | [65] |
| **132 ± 8** | Kidney (FH1) cell culture[h1] | Cyt $bc_1$ redox poise | [66] |
| **155 ± 3** | Kidney (FH1 ko) cell culture[h2] | Cyt $bc_1$ redox poise | [66] |

[a] $\Delta\psi$ is given as positive values here, measured for proton export (from in/N to out/P) as stipulated by Lee. In fact, in the literature most are reported as negative values, for proton import.

[b] work load conditions/metabolite: (1) low/11 mM glucose; (2) medium/11 mM glucose; (3) high/11 mM glucose; (4) low/5 mM lactate; (5) medium/5 mM lactate

[c] metabolite: (1) 5.5 mM glucose; (2) 25 mM glucose; such high [glucose] has been shown to support fermentation rather than OxPhos, thus this value is discarded

[d] extensive calibration methods employed to account for fluorophore binding, aggregation, etc.; (1) normal cells; (2) $Ca^{2+}$-stimulated, low ATP demand

[e] high-resolution fluorescence microscopy; methods employed to account for fluorophore binding, aggregation, etc.; (1) cristae; (2) Inner boundary membrane (IBM)

[f] (1) control, ΔpH = 0.45 ± 0.05; (2) + oligomycin, ΔpH = 0.29 ± 0.05

[g] (1) control, ΔpH = 0.26; (2) + oligomycin, ΔpH = 0.32

[h] (1) FH1 proficient cells, ΔpH = 0.68 ± 0.11; (2) FH1-deficient cells, ΔpH = 0.29 ± 0.03

[k] 16 mM glucose; (1) average of three single cells; [glucose] = 3 mM (2); 5.6 mM (3); 8 mM (4); 12 mM (5); and 16 mM (6).

[m] low $K^+$ medium; in the presence of oligomycin, ouabain, or high $K^+$ medium, $\Delta\psi$ = 170 –173 mV

*Table S1B: isolated mitochondria,* ***in vitro***

| $\Delta\psi$ (mV)[a] | source | method | Ref. |
|---|---|---|---|
| 123.0 ± 1.3 | isolated mitochondria | [$^3$H]$TPP^+$ distribution | 54 |
| 150 | isolated mitochondria | $^{86}Rb^+$ distribution | 18 |
| 145 | isol. Mitoch., state 3 | Safranine $O^+$ fluor. Quench | 67 |
| 170 | isol. Mitoch., state 4 | Safranine $O^+$ fluor. Quench | 67 |
| 145 | isol. Mitoch., state 3 | Safranine $O^+$ fluor. Quench | 68 |
| 170 | isol. Mitoch., state 4 | Safranine $O^+$ fluor. Quench | 68 |
| 172 ± 4 | Isolated mitochondria | $TMRM^+$ fluorescence | 34 |
| 141 | isol. Mitoch., state 3 | [$^3$H]TP(M)$P^+$ distribution | 69 |
| 171 ± 6 | isol. Mitoch., st.3, $K^+$ depleted | $K^+$ distribn./$K^+$ electrode | 70 |
| 139 ± 3 | isol. Mitoch., state 4 | $K^+$ distribn./$K^+$ electrode | 70 |
| 140 | isolated mitoch., lo $O_2$ cons. | $Ph_3MP^+$ distribn/electrode | 71 |
| 180 | isolated mitoch., lo $O_2$ cons. | $Ph_3MP^+$ distribn/electrode | 71 |
| 171 ± 7 | isol. Mitoch., state 1 | $TPP^+$ distributn./electrode | 72 |
| 149 ± 9 | isol. Mitoch., state 2 | $TPP^+$ distributn./electrode | 72 |
| 180 ± 7 | isol. Mitoch., state 3 | $TPP^+$ distributn./electrode | 72 |
| 180 ± 8 | isol. Mitoch., state 4 | $TPP^+$ distributn./electrode | 72 |
| 141 ± 4 | isol. Mitoch., state 3[j1] | $TPMP^+$ distributn./electrode | 73 |
| 139 ± 4 | isol. Mitoch., state 3[j2] | $TPMP^+$ distributn./electrode | 73 |
| 147 ± 3 | isol. Mitoch., state 3[j3] | $TPMP^+$ distributn./electrode | 73 |
| 144.4 ± 0.6 | Isolated mitochondria, wt | [$^3$H]$TPMP^+$ distrib/MitoClick | 74 |
| 152.8 ± 0.6 | Isolated heart mitochondria, complex I-inhibited mut. | [$^3$H]$TPMP^+$ distrib/MitoClick | 74 |
| 180.3 ± 0.9 | Isolated mitochondria, wt, liver, young mice | $TPP^+$ distributn./electrode | 75 |
| 176.7 ± 2.7 | Isolated mitochondria, SOD(+/-), liver, young mice | $TPP^+$ distributn./electrode | 75 |
| 180.4 ± 1.7 | Isolated mitochondria, wt, liver, middle-aged mice | $TPP^+$ distributn./electrode | 75 |
| 176.2 ± 2.2 | Isolated mitochondria, SOD(+/-), liver, mid-age mice | $TPP^+$ distributn./electrode | 75 |
| 169.3 ± 2.5 | Isolated mitochondria, wt, liver, old mice | $TPP^+$ distributn./electrode | 75 |
| 165.5 ± 1.3 | Isolated mitochondria, SOD(+/-), liver, old mice | $TPP^+$ distributn./electrode | 75 |

[j] metabolite = succinate + (1) no addition; (2) rotenone; (3) piericidin

*Meta-analysis (1969-2021) of mitochondrial transmembrane potentials*

Using the distribution of radio-isotopes of amphiphilic cations (e.g., $TPP^+$, $TPMP^+$) to determine $\Delta\psi$ presents experimental difficulties, especially in vivo, where the probed volume also includes interstitial space, vasculature, etc. To distinguish the mitochondrial matrix from the cytoplasm at low spatial resolution (e.g., ≥ 1 μm) is also extremely challenging. Finally, cell culture measurements using fluorophores (e.g., $TMRE^+$, $TMRM^+$) must take into account aggregation, non-specific binding, changes in fluorescence, and other complicating factors. Many of these complexities are discussed by Zorova et al.[76] Besides experimental artifacts, low mitochondrial potential can be caused by mitochondrial dysfunction or metabolic state (e.g., state 4 vs. state 3, open permeability transition pore).[77]

The most reliable fluorescence measurements[58,61,62] employ extensive calibration methods, control experiments, and in the case of Wolf et al, high spatial resolution. The measurements from the Springett group[63,64,65,66] are also reliable because they do not employ an extrinsic sensor, but rather the intrinsic sensor of cytochrome $bc_1$ redox poise. Note that in vivo, these reliable values (132 – 183 mV, bolded in Table S1A) are higher than the others (81 – 125 mV). The experimental difficulties outlined above for in vivo measurements are less prevalent in vitro using isolated mitochondria (Table S1B), where only three aqueous phases are present: matrix, intermembrane space/cristae lumen, and external medium.[76]

Comparing the 24 reliable in vivo $\Delta\psi$ values (bolded in Table S1A) to those measured in vitro (isolated mitochondria, Table S1B), we see no statistical difference: 158 ± 14 mV in

vivo (range: 132 to 180 mV) vs. 160 ± 18 mV in vitro (range: 123 to 180 mV). Averaging both in vivo and in vitro results, we get 159 ± 16 mV; this accords with the typical "normal" potential of 150 mV employed by Nicholls.[78,62] Clearly, the low values (56 to 114 mV) cited by Lee[79] are unreliable; for example, it has been pointed out that below ≈125 mV, the $F_1F_o$ would function in reverse, hydrolyzing rather than synthesizing ATP.[80] From the reliable reported values, we can define the following range of mitochondrial potentials that support oxidative phosphorylation (i.e., ATP synthesis in the matrix on $F_1$): **low-potential (123 mV), average-potential (159 mV), and high-potential (180 mV) mitochondria**. This matches the normal range of mitochondrial $\Delta\psi$ = 120 – 180 mV cited by Logan et al[74], and 130 – 180 mV in Rottenberg's recent meta-analysis.[77]

*V. Literature values for biological membrane specific capacitance*

**Table S2:**

| **Membrane** | **Avg. *C/A*** (μF/cm²) | ***C/A* range** (μF/cm²) | **Reference** |
|---|---|---|---|
| Egg PtdChol | 0.46 | | [81] |
| various PL bilayers | 0.65 ± 0.05 | 0.60 – 0.72 | [82] |
| mitoch. membrane | 0.55 ± 0.05 | 0.5 – 0.6 | [83] |
| chromaffin cell (P.M.)* | 0.5 | | [84] |
| nerve endings | 0.8 | | [85] |
| various membr. in vivo | ≈ 1 | | [86,87] |
| neuron | 0.94 ± 0.20 | 0.8 – 1.3 | [88] |
| mitoch. membrane | 1.2 ± 0.1 | 1.1 – 1.3 | [89] |
| white blood cell | 0.98 ± 0.10 | 0.90 – 0.11 | [90] |
| myeloid leukemia cell* | 1.20 ± 0.14 | | [91] |
| promyelocytic leukemia* | 1.45 ± 0.18 | | [91] |
| lymphoma cell* | 1.40 ± 0.12 | 1.30 – 1.54 | [90] |
| pancreatic cancer cell* | 2.1 ± 0.3 | 1.7 – 2.5 | [90] |

*P.M.: *C/A* for all whole cells measured at the plasma membrane (P.M.)

Note that *C/A* is significantly higher for cancer cell plasma membranes than for other biological membranes.

## VI. *Experimental Measurements of pH at the Membrane Surface*

Lee has employed his TELP hypothesis to predict low values of $pH_{P,surface}$, however, he has not compared these predictions to relevant literature values such as those that have been summarized recently[92] and others that are presented in **Table S3**.

**Table S3**: pH values reported within 1 nm of a water/hydrophobic interface, either measured experimentally with lipid-fluorophore proton sensors or calculated from molecular dynamics simulations or electrostatics. Adapted and expanded with permission from ref. [93], © 2022, Springer-Nature Publishing.

| system | $pH_{surface}$ | method | reference |
|---|---|---|---|
| Lipid bilayer | 5.0 ± 0.2 | lipid fluorophore | 46 |
| Lipid bilayer | 5.0 | lipid fluorophore | 43 |
| Lipid bilayer | 5.5 | lipid fluorophore | 94 |
| mitochondria | 5.9 | lipid fluorophore | 95 |
| Lipid bilayer | 4.7 | Molecular dynamics | 96 |
| Lipid bilayer | 3.3 | Molecular dynamics | 97 |
| Bioenergetic membrane | 6.0 ± 0.5 | electrostatics | 44 |
| Bioenergetic membrane | 6.1 ± 0.2 | electrostatics | 45 |
| Bioenergetic membrane | ≤ 6 | electrostatics | 98 |
| Water/decane interface | 4.4 | Molecular dynamics | 99 |
| Water/hydrophobic | 5.2 | Molecular dynamics | 100 |
| Water/$CHCl_3$ interface | 5.8 | Molecular dynamics | 100 |
| Water/air interface | 5.7 | Molecular dynamics | 100 |
| Water/air interface | 4.8 | Molecular dynamics | 101 |
| Water/air interface | 5.2 | Molecular dynamics | 102 |
| Water/air interface | 6.0 | Molecular dynamics | 103 |
| Water/air interface | 6.5 | Molecular dynamics | 104 |
| Water/air interface | 6.6 | Molecular dynamics | 105 |
| median | 5.4 ± 0.4 | | |

Although these values of surface pH within 1 nm of the water/hydrophobic interface were measured at eight different interfaces, 15 of the 18 reported values cluster between pH 4.4 and 6.1; the median surface pH for all 17 reported values is 5.4 ± 0.4 (± one standard deviation). Based on this range, it seems safe to conclude that the surface pH lies between ≈ 4.5 and 6.5. This is dramatically less acidic than the pH ≈ 2 values predicted by Lee's TELP hypothesis; not one of these papers reported a surface pH close to 2. Thus, literature reports do not support the predictions of the TELP hypothesis.

*VII. Proton uptake and release sites in Complex IV (cytochrome c oxidase)*

**Table S4:**

| **Proton site** | **Channel** | **Residue** | **Distance to HG (nm)*** | **Distance to f.a. (nm)*** |
|---|---|---|---|---|
| inlet | D | $Asp_{91}$ | 0.22 – 0.35 | 1.3 – 1.4 |
| inlet | K | $Glu_{62}$ | 0.5 – 0.9 | 1.6 – 2.0 |
| inlet? | K | $Asp_{57}$ | 0.075 – 0.035 | 1.2 – 1.4 |
| outlet | H | $Asp_{51}$ | ≈ 0.1 | ≈ 1.2 |
| outlet | D, K | $Asp_{173}$ ($glu_{126}$) | ? | ? |

* HG = lipid head group surface at water interface; f.a. = fatty acid/HG interface; calculated using HG layer thickness = 1.1 nm.[41] Variation in the distances is due to differences in the modeled lipid. Distances from Jiapeng Zhu, personal communication, and from ref. [106].

## References


(1) Silverstein, T. P. An Exploration of How the Thermodynamic Efficiency of Bioenergetic Membrane Systems Varies with C-Subunit Stoichiometry of F1F0-ATP Synthases. *J. Bioenerg. Biomembr.* **2014**, *46* (3), 229–241. http://dx.doi.org/10.1007/s10863-014-9547-y.
(2) Lee, J. W. Energy Renewal: Isothermal Utilization of Environmental Heat Energy with Asymmetric Structures. *Entropy* **2021**, *23* (6), 665.
(3) Lee, J. W. Mitochondrial Energetics with Transmembrane Electrostatically Localized Protons: Do We Have a Thermotrophic Feature? *Sci. Rep.* **2021**, *11* (1), 1–13.
(4) Cockrell, R. S.; Harris, E. J.; Pressman, B. C. Energetics of Potassium Transport in Mitochondria Induced by Valinomycin. *Biochemistry* **1966**, *5* (7), 2326–2335. https://doi.org/10.1021/bi00871a022.
(5) Silverstein, T. P. The Real Reason Why ATP Hydrolysis Is Spontaneous at pH > 7: It’s (Mostly) the Proton Concentration! *Biochem. Molec. Biol. Educ.* **2023**, *51* (5), 476–485. https://doi.org/10.1002/bmb.21745.
(6) Rieger, B.; Arroum, T.; Borowski, M.; Villalta, J.; Busch, K. B. Mitochondrial F $_1$ F $_0$ ATP Synthase Determines the Local Proton Motive Force at Cristae Rims. *EMBO Rep.* **2021**, *22* (12), e52727. https://doi.org/10.15252/embr.202152727.
(7) Alberty, R. A.; Goldberg, R. N. Standard Thermodynamic Formation Properties for the Adenosine 5’-Triphosphate Series. *Biochemistry* **1992**, *31* (43), 10610–10615.

(8) Gajewski, E.; Steckler, D. K.; Goldberg, R. N. Thermodynamics of the Hydrolysis of Adenosine 5'-Triphosphate to Adenosine 5'-Diphosphate. *J. Biol. Chem.* **1986**, *261* (27), 12733–12737.
(9) Turina, P. H+/ATP Ratio of Proton Transport-Coupled ATP Synthesis and Hydrolysis Catalysed by CF0F1-Liposomes. *The EMBO Journal* **2003**, *22* (3), 418–426. https://doi.org/10.1093/emboj/cdg073.
(10) Petersen, J.; Förster, K.; Turina, P.; Gräber, P. Comparison of the H $^+$ /ATP Ratios of the H $^+$ -ATP Synthases from Yeast and from Chloroplast. *Proc. Natl. Acad. Sci. U.S.A.* **2012**, *109* (28), 11150–11155. https://doi.org/10.1073/pnas.1202799109.
(11) Soga, N.; Kimura, K.; Kinosita, K.; Yoshida, M.; Suzuki, T. Perfect Chemomechanical Coupling of F $_o$ F $_1$ -ATP Synthase. *Proc. Natl. Acad. Sci. U.S.A.* **2017**, *114* (19), 4960–4965. https://doi.org/10.1073/pnas.1700801114.
(12) Steigmiller, S.; Turina, P.; Gräber, P. The Thermodynamic H $^+$ /ATP Ratios of the H $^+$ -ATPsynthases from Chloroplasts and *Escherichia Coli*. *Proc. Natl. Acad. Sci. U.S.A.* **2008**, *105* (10), 3745–3750. https://doi.org/10.1073/pnas.0708356105.
(13) Gyulai, L.; Roth, Z.; Leigh Jr, J. S.; Chance, B. Bioenergetic Studies of Mitochondrial Oxidative Phosphorylation Using 31phosphorus NMR. *J. Biol. Chem.* **1985**, *260* (7), 3947–3954.
(14) Forman, N. G.; Wilson, D. F. Dependence of Mitochondrial Oxidative Phosphorylation on Activity of the Adenine Nucleotide Translocase. *J. Biol. Chem.* **1983**, *258* (14), 8649–8655.
(15) Ackerman, J. J.; Grove, T. H.; Wong, G. G.; Gadian, D. G.; Radda, G. K. Mapping of Metabolites in Whole Animals by 31P NMR Using Surface Coils. *Nature* **1980**, *283* (5743), 167–170.
(16) Jacobus, W. E.; Moreadith, R. W.; Vandegaer, K. M. Mitochondrial Respiratory Control. Evidence against the Regulation of Respiration by Extramitochondrial Phosphorylation Potentials or by [ATP]/[ADP] Ratios. *J. Biol. Chem.* **1982**, *257* (5), 2397–2402.
(17) Nioka, S.; Chance, B.; Hilberman, M.; Subramanian, H. V.; Leigh, J. S.; Veech, R. L.; Forster, R. E. Relationship between Intracellular pH and Energy Metabolism in Dog Brain as Measured by 31P-NMR. *J. Appl. Physiol.* **1987**, *62* (5), 2094–2102. https://doi.org/10.1152/jappl.1987.62.5.2094.
(18) Nicholls, D. G. The Influence of Respiration and ATP Hydrolysis on the Proton-Electrochemical Gradient across the Inner Membrane of Rat-Liver Mitochondria as Determined by Ion Distribution. *Eur. J. Biochem.* **1974**, *50* (1), 305–315.
(19) Heldt, H. W.; Klingenberg, M.; Milovancev, M. Differences between the ATP/ADP Ratios in the Mitochondrial Matrix and in the Extramitochondrial Space. *European Journal of Biochemistry* **1972**, *30* (3), 434–440. https://doi.org/10.1111/j.1432-1033.1972.tb02115.x.
(20) Veech, R. L.; Lawson, J. W.; Cornell, N. W.; Krebs, H. A. Cytosolic Phosphorylation Potential. *J. Biol. Chem.* **1979**, *254* (14), 6538–6547.
(21) Davis, E. J.; Lumeng, L. Relationships between the Phosphorylation Potentials Generated by Liver Mitochondria and Respiratory State under Conditions of Adenosine Diphosphate Control. *J. Biol. Chem.* **1975**, *250* (6), 2275–2282.
(22) Kohn, M. C.; Achs, M. J.; Garfinkel, D. Distribution of Adenine Nucleotides in the Perfused Rat Heart. *Amer. J. Physiol. - Reg. Integr. Comp. Physiol* **1977**, *232* (5), R158–R163. https://doi.org/10.1152/ajpregu.1977.232.5.R158.
(23) Akerboom, T. P. M.; Bookelman, H.; Zuurendonk, P. F.; Meer, R.; Tager, J. M. Intramitochondrial and Extramitochondrial Concentrations of Adenine Nucleotides

and Inorganic Phosphate in Isolated Hepatocytes from Fasted Rats. *Eur. J. Biochem.* **1978**, *84* (2), 413–420. https://doi.org/10.1111/j.1432-1033.1978.tb12182.x.
(24) Steenbergen, C.; Deleeuw, G.; Rich, T.; Williamson, J. R. Effects of Acidosis and Ischemia on Contractility and Intracellular pH of Rat Heart. *Circ. Res.* **1977**, *41* (6), 849–858. https://doi.org/10.1161/01.RES.41.6.849.
(25) Bohnensack, R. Control of Energy Transformation in Mitochondria. Analysis by a Quantitative Model. *Biochim. Biophys. Acta - Bioenerg.* **1981**, *634*, 203–218.
(26) Letko, G.; Küster, U.; Duszyński, J.; Kunz, W. Investigation of the Dependence of the Intramitochondrial [ATP]/[ADP] Ratio on the Respiration Rate. *Biochim. Biophys. Acta - Bioenerg.* **1980**, *593* (2), 196–203.
(27) Küster, U.; Letko, G.; Kunz, W.; Duszyńsky, J.; Bogucka, K.; Wojtczak, L. Influence of Different Energy Drains on the Interrelationship between the Rate of Respiration, Proton-Motive Force and Adenine Nucleotide Patterns in Isolated Mitochondria. *Biochim. Biophys. Acta - Bioenerg.* **1981**, *636* (1), 32–38.
(28) Stitt, M.; Lilley, R. M.; Heldt, H. W. Adenine Nucleotide Levels in the Cytosol, Chloroplasts, and Mitochondria of Wheat Leaf Protoplasts. *Plant Physiol.* **1982**, *70* (4), 971–977.
(29) Gout, E.; Rébeillé, F.; Douce, R.; Bligny, R. Interplay of $Mg^{2+}$, ADP, and ATP in the Cytosol and Mitochondria: Unravelling the Role of $Mg^{2+}$ in Cell Respiration. *Proc. Natl. Acad. Sci. U.S.A.* **2014**, *111* (43). https://doi.org/10.1073/pnas.1406251111.
(30) Silverstein, T. P. The Proton in Biochemistry: Impacts on Bioenergetics, Biophysical Chemistry, and Bioorganic Chemistry. *Front. Mol. Biosci.* **2021**, *8*, 1033. https://doi.org/10.3389/fmolb.2021.764099.
(31) McCullagh, M.; Saunders, M. G.; Voth, G. A. Unraveling the Mystery of ATP Hydrolysis in Actin Filaments. *J. Am. Chem. Soc.* **2014**, *136* (37), 13053–13058. https://doi.org/10.1021/ja507169f.
(32) McCullagh, M.; Saunders, M. G.; Voth, G. A. Unraveling the Mystery of ATP Hydrolysis in Actin Filaments. *Biophys. J.* **2015**, *108* (2), 297a.
(33) Kudryashov, D. S.; Reisler, E. ATP and ADP Actin States. *Biopolymers* **2013**, *99* (4), 245–256. https://doi.org/10.1002/bip.22155.
(34) Wei, A.-C.; Liu, T.; Winslow, R. L.; O'Rourke, B. Dynamics of Matrix-Free Ca2+ in Cardiac Mitochondria: Two Components of Ca2+ Uptake and Role of Phosphate Buffering. *J. Gen. Physiol.* **2012**, *139* (6), 465–478.
(35) Kushnareva, Y. E.; Haley, L. M.; Sokolove, P. M. The Role of Low (≤ 1 mM) Phosphate Concentrations in Regulation of Mitochondrial Permeability: Modulation of Matrix Free Ca2+ Concentration. *Arch. Biochem. Biophys.* **1999**, *363* (1), 155–162.
(36) Nicholls, D. G. Mitochondrial Membrane Potential and Aging. *Aging cell* **2004**, *3* (1), 35–40.
(37) Nicholls, D. G. Mitochondria and Calcium Signaling. *Cell calcium* **2005**, *38* (3–4), 311–317.
(38) Gardestrom, P. E. R.; Wigge, B. Influence of Photorespiration on ATP/ADP Ratios in the Chloroplasts, Mitochondria, and Cytosol, Studied by Rapid Fractionation of Barley (Hordeum Vulgare) Protoplasts. *Plant Physiol.* **1988**, *88* (1), 69–76.
(39) Böhme, G.; Hartung, K. J.; Kunz, W. Bioenergetics at Mitochondrial and Cellular Levels. In *Bioenergetics at Mitochondrial and Cellular Levels (Wojtczak, L.,*

*Lenartowicz, E. and Zborowski, J., eds.)*; Nencki Institute of Experimental Biology: Warsaw, 1978; pp 79–102.
(40) Klingenberg, M. The ADP and ATP Transport in Mitochondria and Its Carrier. *Biochim. Biophys. Acta - Biomembr.* **2008**, *1778* (10), 1978–2021.
(41) Silverstein, T. P. Is Localized Chemiosmosis Necessary in Mitochondria? Is Lee's Protonic Capacitor Hypothesis a Valid Model? *Mitochondrial Commun.* **2024**, *2*, 48–57. https://doi.org/10.1016/j.mitoco.2024.06.001.
(42) Silverstein, T. P. Lee's "Transmembrane Electrostatically-Localized Proton" Model Does NOT Offer a Better Understanding of Neuronal Transmembrane Potentials. *J. Neurophysiol.* **2023**, *130* (1), 123–127. https://doi.org/10.1152/jn.00173.2023.
(43) Tocanne, J.-F.; Teissié, J. Ionization of Phospholipids and Phospholipid-Supported Interfacial Lateral Diffusion of Protons in Membrane Model Systems. *Biochim. Biophys. Acta - Rev. Biomembr.* **1990**, *1031* (1), 111–142.
(44) Mulkidjanian, A. Y.; Heberle, J.; Cherepanov, D. A. Protons@ Interfaces: Implications for Biological Energy Conversion. *Biochim. Biophys. Acta - Bioenerg.* **2006**, *1757* (8), 913–930.
(45) Cherepanov, D. A.; Feniouk, B. A.; Junge, W.; Mulkidjanian, A. Y. Low Dielectric Permittivity of Water at the Membrane Interface: Effect on the Energy Coupling Mechanism in Biological Membranes. *Biophys. J.* **2003**, *85* (2), 1307–1316.
(46) Weichselbaum, E.; Österbauer, M.; Knyazev, D. G.; Batishchev, O. V.; Akimov, S. A.; Hai Nguyen, T.; Zhang, C.; Agmon, N.; Carloni, P.; Pohl, P.; et al. Origin of Proton Affinity to Membrane/Water Interfaces. *Sci. Rep.* **2017**, *7* (1), 4553.
(47) Nguyen, T. H.; Zhang, C.; Weichselbaum, E.; Knyazev, D. G.; Pohl, P.; Carloni, P. Interfacial Water Molecules at Biological Membranes: Structural Features and Role for Lateral Proton Diffusion. *PLoS One* **2018**, *13* (2), e0193454.
(48) Wan, B.; Doumen, C.; Duszynski, J.; Salama, G.; Vary, T. C.; LaNoue, K. F. Effects of Cardiac Work on Electrical Potential Gradient across Mitochondrial Membrane in Perfused Rat Hearts. *Amer. J. Physiol. - Heart* **1993**, *265* (2), H453–H460. https://doi.org/10.1152/ajpheart.1993.265.2.H453.
(49) Gurm, G. S.; Danik, S. B.; Shoup, T. M.; Weise, S.; Takahashi, K.; Laferrier, S.; Elmaleh, D. R.; Gewirtz, H. 4-[18F]-Tetraphenylphosphonium as a PET Tracer for Myocardial Mitochondrial Membrane Potential. *JACC: Cardiovasc. Imag.* **2012**, *5* (3), 285–292. https://doi.org/10.1016/j.jcmg.2011.11.017.
(50) Alpert, N. M.; Guehl, N.; Ptaszek, L.; Pelletier-Galarneau, M.; Ruskin, J.; Mansour, M. C.; Wooten, D.; Ma, C.; Takahashi, K.; Zhou, Y. Quantitative in Vivo Mapping of Myocardial Mitochondrial Membrane Potential. *PloS one* **2018**, *13* (1), e0190968.
(51) Pelletier-Galarneau, M.; Petibon, Y.; Ma, C.; Han, P.; Kim, S. J. W.; Detmer, F. J.; Yokell, D.; Guehl, N.; Normandin, M.; El Fakhri, G.; Alpert, N. M. In Vivo Quantitative Mapping of Human Mitochondrial Cardiac Membrane Potential: A Feasibility Study. *Eur J Nucl Med Mol Imaging* **2021**, *48* (2), 414–420. https://doi.org/10.1007/s00259-020-04878-9.
(52) Kauppinen, R. A.; Hassinen, I. E. Monitoring of Mitochondrial Membrane Potential in Isolated Perfused Rat Heart. *Amer. J. Physiol. - Heart* **1984**, *247* (4), H508–H516. https://doi.org/10.1152/ajpheart.1984.247.4.H508.
(53) Zhang, H.; Huang, H. M.; Carson, R. C.; Mahmood, J.; Thomas, H. M.; Gibson, G. E. Assessment of Membrane Potentials of Mitochondrial Populations in Living Cells. *Anal. Biochem.* **2001**, *298* (2), 170–180.

(54) LaNoue, K. F.; Strzelecki, T.; Strzelecka, D.; Koch, C. Regulation of the Uncoupling Protein in Brown Adipose Tissue. *J. Biol. Chem.* **1986**, *261* (1), 298–305.
(55) Hagen, T. M.; Yowe, D. L.; Bartholomew, J. C.; Wehr, C. M.; Do, K. L.; Park, J.-Y.; Ames, B. N. Mitochondrial Decay in Hepatocytes from Old Rats: Membrane Potential Declines, Heterogeneity and Oxidants Increase. *Proc. Natl. Acad. Sci. U.S.A.* **1997**, *94* (7), 3064–3069. https://doi.org/10.1073/pnas.94.7.3064.
(56) Hoek, J. B.; Nicholls, D. G.; Williamson, J. R. Determination of the Mitochondrial Protonmotive Force in Isolated Hepatocytes. *J. Biol. Chem.* **1980**, *255* (4), 1458–1464.
(57) Scott, I. D.; Nicholls, D. G. Energy Transduction in Intact Synaptosomes. Influence of Plasma-Membrane Depolarization on the Respiration and Membrane Potential of Internal Mitochondria Determined in Situ. *Biochem. J.* **1980**, *186* (1), 21–33.
(58) Gerencser, A. A.; Chinopoulos, C.; Birket, M. J.; Jastroch, M.; Vitelli, C.; Nicholls, D. G.; Brand, M. D. Quantitative Measurement of Mitochondrial Membrane Potential in Cultured Cells: Calcium-induced De- and Hyperpolarization of Neuronal Mitochondria. *J. Physiol.* **2012**, *590* (12), 2845–2871. https://doi.org/10.1113/jphysiol.2012.228387.
(59) Gerencser, A. A.; Mookerjee, S. A.; Jastroch, M.; Brand, M. D. Measurement of the Absolute Magnitude and Time Courses of Mitochondrial Membrane Potential in Primary and Clonal Pancreatic Beta-Cells. *PLoS One* **2016**, *11* (7), e0159199.
(60) Lerner, C. A.; Gerencser, A. A. Unbiased Millivolts Assay of Mitochondrial Membrane Potential in Intact Cells. In *Mitochondria*; Tomar, N., Ed.; Methods in Molecular Biology; Springer US: New York, NY, 2022; Vol. 2497, pp 11–61. https://doi.org/10.1007/978-1-0716-2309-1_2.
(61) Wolf, D. M.; Segawa, M.; Kondadi, A. K.; Anand, R.; Bailey, S. T.; Reichert, A. S.; Van Der Bliek, A. M.; Shackelford, D. B.; Liesa, M.; Shirihai, O. S. Individual Cristae within the Same Mitochondrion Display Different Membrane Potentials and Are Functionally Independent. *EMBO J.* **2019**, *38* (22), e101056. https://doi.org/10.15252/embj.2018101056.
(62) Nicholls, D. G. Simultaneous Monitoring of Ionophore-and Inhibitor-Mediated Plasma and Mitochondrial Membrane Potential Changes in Cultured Neurons. *J. Biol. Chem.* **2006**, *281* (21), 14864–14874.
(63) Kim, N.; Ripple, M. O.; Springett, R. Measurement of the Mitochondrial Membrane Potential and pH Gradient from the Redox Poise of the Hemes of the Bc1 Complex. *Biophys. J.* **2012**, *102* (5), 1194–1203.
(64) Ripple, M. O.; Kim, N.; Springett, R. Mammalian Complex I Pumps 4 Protons per 2 Electrons at High and Physiological Proton Motive Force in Living Cells. *J. Biol. Chem.* **2013**, *288* (8), 5374–5380.
(65) Aras, S.; Bai, M.; Lee, I.; Springett, R.; Hüttemann, M.; Grossman, L. I. MNRR1 (Formerly CHCHD2) Is a Bi-Organellar Regulator of Mitochondrial Metabolism. *Mitochondrion* **2015**, *20*, 43–51.
(66) Tyrakis, P. A.; Yurkovich, M. E.; Sciacovelli, M.; Papachristou, E. K.; Bridges, H. R.; Gaude, E.; Schreiner, A.; D’Santos, C.; Hirst, J.; Hernandez-Fernaud, J. Fumarate Hydratase Loss Causes Combined Respiratory Chain Defects. *Cell Rep.* **2017**, *21* (4), 1036–1047.

(67) Chinopoulos, C.; Vajda, S.; Csanády, L.; Mándi, M.; Mathe, K.; Adam-Vizi, V. A Novel Kinetic Assay of Mitochondrial ATP-ADP Exchange Rate Mediated by the ANT. *Biophys. J.* **2009**, *96* (6), 2490–2504.
(68) Metelkin, E.; Demin, O.; Kovács, Z.; Chinopoulos, C. Modeling of ATP–ADP Steady-state Exchange Rate Mediated by the Adenine Nucleotide Translocase in Isolated Mitochondria. *FEBS J.* **2009**, *276* (23), 6942–6955. https://doi.org/10.1111/j.1742-4658.2009.07394.x.
(69) Rottenberg, H. Membrane Potential and Surface Potential in Mitochondria: Uptake and Binding of Lipophilic Cations. *J. Membr. Biol.* **1984**, *81* (2), 127–138. https://doi.org/10.1007/BF01868977.
(70) Mitchell, P.; Moyle, J. Estimation of Membrane Potential and pH Difference across the Cristae Membrane of Rat Liver Mitochondria. *Eur. J. Biochem.* **1969**, *7* (4), 471–484. https://doi.org/10.1111/j.1432-1033.1969.tb19633.x.
(71) Hafner, R. P.; Brown, G. C.; Brand, M. D. Analysis of the Control of Respiration Rate, Phosphorylation Rate, Proton Leak Rate and Protonmotive Force in Isolated Mitochondria Using the "top-down" Approach of Metabolic Control Theory. *Eur. J. Biochem.* **1990**, *188* (2), 313–319. https://doi.org/10.1111/j.1432-1033.1990.tb15405.x.
(72) Kamo, N.; Muratsugu, M.; Hongoh, R.; Kobatake, Y. Membrane Potential of Mitochondria Measured with an Electrode Sensitive to Tetraphenyl Phosphonium and Relationship between Proton Electrochemical Potential and Phosphorylation Potential in Steady State. *J. Membr. Biol.* **1979**, *49* (2), 105–121. https://doi.org/10.1007/BF01868720.
(73) Lambert, A. J.; Brand, M. D. Superoxide Production by NADH: Ubiquinone Oxidoreductase (Complex I) Depends on the pH Gradient across the Mitochondrial Inner Membrane. *Biochem. J.* **2004**, *382* (2), 511–517.
(74) Logan, A.; Pell, V. R.; Shaffer, K. J.; Evans, C.; Stanley, N. J.; Robb, E. L.; Prime, T. A.; Chouchani, E. T.; Cocheme, H. M.; Fearnley, I. M. Assessing the Mitochondrial Membrane Potential in Cells and in Vivo Using Targeted Click Chemistry and Mass Spectrometry. *Cell Metab.* **2016**, *23* (2), 379–385.
(75) Kokoszka, J. E.; Coskun, P.; Esposito, L. A.; Wallace, D. C. Increased Mitochondrial Oxidative Stress in the Sod2 (+/−) Mouse Results in the Age-Related Decline of Mitochondrial Function Culminating in Increased Apoptosis. *Proc. Natl. Acad. Sci. U.S.A.* **2001**, *98* (5), 2278–2283. https://doi.org/10.1073/pnas.051627098.
(76) Zorova, L. D.; Popkov, V. A.; Plotnikov, E. Y.; Silachev, D. N.; Pevzner, I. B.; Jankauskas, S. S.; Babenko, V. A.; Zorov, S. D.; Balakireva, A. V.; Juhaszova, M. Mitochondrial Membrane Potential. *Analytical biochemistry* **2018**, *552*, 50–59.
(77) Rottenberg, H. The Reduction in the Mitochondrial Membrane Potential in Aging: The Role of the Mitochondrial Permeability Transition Pore. *Int. J. Molec. Sci.* **2023**, *24* (15), 12295.
(78) Rego, A. C.; Vesce, S.; Nicholls, D. G. The Mechanism of Mitochondrial Membrane Potential Retention Following Release of Cytochrome c in Apoptotic GT1-7 Neural Cells. *Cell Death & Differentiation* **2001**, *8* (10), 995–1003.
(79) Lee, J. W. TELP Theory: Elucidating the Major Observations of Rieger et al. 2021 in Mitochondria. *Mitoch. Commun.* **2023**, *1*, 62–72.
(80) Chinopoulos, C. Mitochondrial Consumption of Cytosolic ATP: Not so Fast. *FEBS Lett.* **2011**, *585* (9), 1255–1259.

(81) Hanai, T.; Haydon, D. A.; Taylor, J. The Variation of Capacitance and Conductance of Bimolecular Lipid Membranes with Area. *Journal of theoretical biology* **1965**, *9* (3), 433–443.
(82) Gross, L.; Gross, L. C. Applications of Droplet Interface Bilayers: Specific Capacitance Measurements and Membrane Protein Corralling. PhD Thesis, University of Oxford, 2011. https://ora.ox.ac.uk/objects/uuid:0b7ffba6-b86d-499c-a93f-3b2fc46a427b (accessed 2024-01-04).
(83) Pauly, H.; Packer, L.; Schwan, H. P. Electrical Properties of Mitochondrial Membranes. *The Journal of biophysical and biochemical cytology* **1960**, *7* (4), 589.
(84) Solsona, C.; Innocenti, B.; Fernández, J. M. Regulation of Exocytotic Fusion by Cell Inflation. *Biophysical Journal* **1998**, *74* (2), 1061–1073.
(85) Rosenboom, H.; Lindau, M. Exo-Endocytosis and Closing of the Fission Pore during Endocytosis in Single Pituitary Nerve Terminals Internally Perfused with High Calcium Concentrations. *Proc. Natl. Acad. Sci. U.S.A.* **1994**, *91* (12), 5267–5271. https://doi.org/10.1073/pnas.91.12.5267.
(86) Cole, K. S. *Membranes, Ions and Impulses: A Chapter of Classical Biophysics*; Univ of California Press, 1972; Vol. 1.
(87) Neher, E.; Marty, A. Discrete Changes of Cell Membrane Capacitance Observed under Conditions of Enhanced Secretion in Bovine Adrenal Chromaffin Cells. *Proc. Natl. Acad. Sci. U.S.A.* **1982**, *79* (21), 6712–6716. https://doi.org/10.1073/pnas.79.21.6712.
(88) Gentet, L. J.; Stuart, G. J.; Clements, J. D. Direct Measurement of Specific Membrane Capacitance in Neurons. *Biophysical journal* **2000**, *79* (1), 314–320.
(89) Pauly, H.; Packer, L. The Relationship of Internal Conductance and Membrane Capacity to Mitochondrial Volume. *The Journal of Biophysical and Biochemical Cytology* **1960**, *7* (4), 603.
(90) Lannin, T.; Su, W.-W.; Gruber, C.; Cardle, I.; Huang, C.; Thege, F.; Kirby, B. Automated Electrorotation Shows Electrokinetic Separation of Pancreatic Cancer Cells Is Robust to Acquired Chemotherapy Resistance, Serum Starvation, and EMT. *Biomicrofluidics* **2016**, *10* (6).
(91) Zheng, Y.; Shojaei-Baghini, E.; Wang, C.; Sun, Y. Microfluidic Characterization of Specific Membrane Capacitance and Cytoplasm Conductivity of Single Cells. *Biosens. Bioelectr.* **2013**, *42*, 496–502.
(92) Silverstein, T. P. A Critique of the Capacitor-Based "Transmembrane Electrostatically Localized Proton" Hypothesis. *J. Bioenerg. Biomembr.* **2022**, *54* (2), 59–65.
(93) Silverstein, T. P. A Critique of the Capacitor-Based "Transmembrane Electrostatically Localized Proton" Hypothesis. *J. Bioenerg. Biomembr.* **2022**, *54* (2), 59–65. https://doi.org/10.1007/s10863-022-09931-w.
(94) Xu, L.; Öjemyr, L. N.; Bergstrand, J.; Brzezinski, P.; Widengren, J. Protonation Dynamics on Lipid Nanodiscs: Influence of the Membrane Surface Area and External Buffers. *Biophysical journal* **2016**, *110* (9), 1993–2003.
(95) Xiong, J.-W.; Zhu, L.; Jiao, X.; Liu, S.-S. Evidence for ΔpH Surface Component (ΔpHS) of Proton Motive Force in ATP Synthesis of Mitochondria. *Biochim. Biophys. Acta - Gen. Subj.* **2010**, *1800* (3), 213–222.
(96) Wolf, M. G.; Grubmüller, H.; Groenhof, G. Anomalous Surface Diffusion of Protons on Lipid Membranes. *Biophys. J.* **2014**, *107* (1), 76–87.
(97) Yamashita, T.; Voth, G. A. Properties of Hydrated Excess Protons near Phospholipid Bilayers. *J. Phys. Chem. B* **2010**, *114* (1), 592–603.

(98) Mulkidjanian, A.; Cherepanov, D.; Heberle, J.; Junge, W. Proton Transfer Dynamics at Membrane/Water Interface and Mechanism of Biological Energy Conversion. *Biochem. (Moscow)* **2005**, *70* (2), 251–256.
(99) Zhang, C.; Knyazev, D. G.; Vereshaga, Y. A.; Ippoliti, E.; Nguyen, T. H.; Carloni, P.; Pohl, P. Water at Hydrophobic Interfaces Delays Proton Surface-to-Bulk Transfer and Provides a Pathway for Lateral Proton Diffusion. *Proc. Natl. Acad. Sci. U.S.A.* **2012**, *109* (25), 9744–9749.
(100) Iuchi, S.; Chen, H.; Paesani, F.; Voth, G. A. Hydrated Excess Proton at Water-Hydrophobic Interfaces. *J. Phys. Chem. B* **2009**, *113* (13), 4017–4030.
(101) Vácha, R.; Buch, V.; Milet, A.; Devlin, J. P.; Jungwirth, P. Autoionization at the Surface of Neat Water: Is the Top Layer pH Neutral, Basic, or Acidic? *Phys. Chem. Chem. Phys.* **2007**, *9* (34), 4736–4747.
(102) Köfinger, J.; Dellago, C. Biasing the Center of Charge in Molecular Dynamics Simulations with Empirical Valence Bond Models: Free Energetics of an Excess Proton in a Water Droplet. *J. Phys. Chem. B* **2008**, *112* (8), 2349–2356.
(103) Lee, H.-S.; Tuckerman, M. E. Ab Initio Molecular Dynamics Studies of the Liquid- Vapor Interface of an HCl Solution. *J. Phys. Chem. A* **2009**, *113* (10), 2144–2151.
(104) Wick, C. D.; Kuo, I.-F. W.; Mundy, C. J.; Dang, L. X. The Effect of Polarizability for Understanding the Molecular Structure of Aqueous Interfaces. *J. Chem. Theor. Comput.* **2007**, *3* (6), 2002–2010.
(105) Tse, Y.-L. S.; Chen, C.; Lindberg, G. E.; Kumar, R.; Voth, G. A. Propensity of Hydrated Excess Protons and Hydroxide Anions for the Air–Water Interface. *J. Am. Chem. Soc.* **2015**, *137* (39), 12610–12616.
(106) Noodleman, L.; Götz, A. W.; Du, W.-G. H.; Hunsicker-Wang, L. Reaction Pathways, Proton Transfer, and Proton Pumping in Ba3 Class Cytochrome c Oxidase: Perspectives from DFT Quantum Chemistry and Molecular Dynamics. *Front. Chem.* **2023**, *11*, 1186022. https://doi.org/10.3389/fchem.2023.1186022.
